# Distribution and neuronal expression of phosphatidylinositol phosphate kinase IIγ in the mouse brain

**DOI:** 10.1002/cne.22161

**Published:** 2009-07-16

**Authors:** Jonathan H Clarke, Piers C Emson, Robin F Irvine

**Affiliations:** 1 Department of Pharmacology, University of CambridgeCambridge, CB2 1PD United Kingdom; 2 Laboratory of Molecular Neuroscience, The Babraham InstituteCambridge, CB22 3AT United Kingdom

**Keywords:** phosphoinositide, PtdIns5P 4-kinase, PIP4K, Purkinje, pyramidal, neuronal expression

## Abstract

The role of cellular phosphatidylinositol 5-phosphate (PtdIns5P), as a signalling molecule or as a substrate for the production of small, compartmentalized pools of phosphatidylinositol 4,5-bisphosphate [PtdIns(4,5)P_2_], may be dependent on cell type and subcellular localization. PtdIns5P levels are primarily regulated by the PtdIns5P 4-kinases (type II PIP kinases or PIP4Ks), and we have investigated the expression and localization in the brain of the least-studied PIP4K isoform, PIP4Kγ. In situ hybridization and immunohistochemistry, using antisense oligonucleotide probes and a PIP4Kγ-specific antibody, revealed that this isoform has a restricted CNS expression profile. The use of antibodies to different cell markers showed that this expression is limited to neurons, particularly the cerebellar Purkinje cells, pyramidal cells of the hippocampus, large neuronal cell types in the cerebral cortex including pyramidal cells, and mitral cells in the olfactory bulb and is not expressed in cerebellar, hippocampal formation, or olfactory bulb granule cells. In neurons expressing this enzyme, PIP4Kγ has a vesicular distribution and shows partial colocalization with markers of cellular compartments of the endomembrane trafficking pathway. The PIP4Kγ isoform expression is established after day 7 of postnatal development. Overall, our data suggest that PIP4Kγ may have a role in neuron function, specifically in the regulation of vesicular transport, in specific regions of the developed brain. J. Comp. Neurol. 517:296–312, 2009. © 2009 Wiley-Liss, Inc.

Polyphosphoinositides (PIs) are emerging as important contributors to many physiological functions in cells and, as minor phospholipid components of cellular membranes, have important roles in signalling pathways. These are involved in diverse functions such as cell migration and proliferation, ion channel regulation, and membrane trafficking (for reviews see Di Paolo and De Camilli,^2006^; Gamper and Shapiro,^2007^; Irvine,^2003^; Pendaries et al.,^2005^; Suh and Hille,^2005^; Yin and Janmey,^2003^).

The canonical pathway to the production of phosphatidylinositol 4,5-bisphosphate [(PtdIns(4,5)P_2_] in the cellular PI-cycle involves the 5-kinase activity of the type I PtdInsP kinases (PIP5Ks) using phosphatidylinositol 4-phosphate (PtdIns4P) as substrate (Ishihara et al.,^1998^). However, since the discovery of phosphatidylinositol 5-phosphate (PtdIns5P) in cells (Rameh et al.,^1997^), another potential route has been found via the 4-kinase activity of type II PtdInsP kinases (here referred to as PIP4Ks, consistent with their substrate specificity, although it should be noted that gene nomenclature still refers to them as *PIP5K2s*). The PIP4Ks are present as α, β, and γ isoforms in vertebrates, and there is evidence that each isoform has a specific compartmentalization in cells that could be intrinsic to its function. PIP4Kβ is present in the nucleus (Bunce et al.,^2008^; Ciruela et al.,^2000^; Richardson et al.,^2007^), where it may be involved in stress responses (Gozani et al.,^2003^; Jones et al.,^2006^; Zou et al.,^2007^). PIP4Kα, the most active isoform, is predominantly cytosolic (Hinchliffe et al.,^1999^; Wilcox and Hinchliffe,^2008^) and can be recruited to the plasma membrane by interaction with a PIP5K (Hinchliffe et al.,^2002^).

The regulation of PtdIns(4,5)P_2_ levels in the brain has been associated with numerous diseases, such as cancer, bipolar disorder, and channelopathies (for reviews see Endersby and Baker,^2008^; Halstead et al.,^2005^; Jentsch et al.,^2004^). Phosphoinositide regulation of a wide variety of neuronal ion transporters (Gamper and Shapiro,^2007^; Suh and Hille,^2005^) suggests that aberrant PtdIns(4,5)P_2_ metabolism may be involved in epilepsy and Alzheimer's disease (Jentsch et al.,^2004^; Landman et al.,^2006^) and may contribute to symptoms of Down's syndrome (Voronov et al.,^2008^). Specific genetic mutations have also linked PIP4Kα with schizophrenia (Fedorenko et al.,^2008^), and disruption of the *PIP5K2B* gene is a common feature in neuroblastoma (Schleiermacher et al.,^2005^). All of the PIP4K and PIP5K isoforms are expressed in the brain (Akiba et al.,^2002^), and the PIP5Kγ splice variants have been most extensively studied, having a major role in synaptic vesicle cycling (Di Paolo et al.,^2004^; Nakano-Kobayashi et al.,^2007^; Wenk et al.,^2001^). PIP5K is also involved in neuronal maintenance (Giudici et al.,^2004^), development and migration (Coulson et al.,^2008^; Wang et al.,^2007^), and neurite remodelling (van Horck et al.,^2002^), suggesting that polyphosphoinositides play significant roles in both neurogenesis and neuronal function, including learning and memory. The observed interaction of PIP4K and PIP5K isoforms (Hinchliffe et al.,^2002^) may also result in the recruitment of specific PIP4Ks required to maintain distinct phosphoinositide pools within different cellular compartments.

The PIP4Kγ isoform has not yet been associated with a cellular function and is expressed predominantly in the kidney and brain (Akiba et al.,^2002^; Clarke et al.,^2008^; Itoh et al.,^1998^). In the present study, we have extensively investigated the localization of PIP4Kγ in the brain by using a specific antibody to this isoform and found that it is remarkably confined to specific neuronal populations, where it is localized to vesicular structures. Developmental studies imply that this enzyme may be involved in neuronal function rather than in neurogenesis. Overall, our results suggest that PIP4Kγ may have a specialized function in vesicle trafficking in specific neuronal cells.

## MATERIALS AND METHODS

### PIP4Kγ cloning and expression

The *PIP5K2C* gene was amplified from a whole human brain marathon-ready cDNA library (Clontech, Mountain View, CA) and cloned into the plasmid expression vectors pET-32a (Novagen, Madison, WI) and pEGFP-C1 (Clontech) as previously described (Clarke et al.,^2008^). Full-length recombinant PIP4Kγ was obtained by enterokinase cleavage of protein purified by TALON metal affinity resin (Clontech) from cell culture lysates of *Escherichia coli* BL21(DE3)pLysS transformed with the bacterial expression construct.

Endotoxin-free pEGFP-C1 plasmid constructs, purified from bacterial clones (Qiagen purification kit, Huntsville, AL), were used for eukaryotic expression. HeLa cells, maintained in DMEM (Gibco, Paisley, United Kingdom) supplemented with 10% fetal bovine serum, 50 U/ml penicillin, and 50 μg/ml streptomycin were transiently transfected for 24 hours using TransFectin reagent (Bio-Rad, Hercules, CA), following the manufacturer's protocol.

### Tissue preparation

Animal care was in accordance with institutional and national guidelines, and all procedures were performed in accordance with Home Office guidelines, Animals (Scientific Procedures) Act of 1986 under Home Office Project licence 80/1747. Samples were collected from P1, P7, P14, P21, P28, and adult male CD1 mice. Tissues used for cDNA library construction and Western blotting lysates were collected post-mortem from six animals and immediately frozen on dry ice. For imaging experiments, brains were removed from three mice that had been terminally anesthetized intraperitoneally with sodium pentobarbital and perfused transcardially with phosphate-buffered saline followed by 4% paraformaldehyde (PFA) in 0.1 M phosphate buffer, pH 7.4. After cryoprotection in 0.1 M phosphate buffer with 30% sucrose, brains were stored at –80°C.

### RT-PCR and in situ hybridization

Full-length mRNA was extracted as described previously (Clarke et al.,^2008^), and cDNA libraries were made for each tissue by reverse transcription (Sprint Powerscript kit; Clontech). PCR amplification was performed with Taq DNA polymerase (Invitrogen, Paisley, United Kingdom) and primers specific to *PIP5K2A* (forward: 5′-AAGAGTCTGATGCCAAGAACCTGT-3′ and reverse: 5′-TGCAGTGCAACTTAAGGATGGTAA-3′), *PIP5K2B* (forward: 5′-CATCCTCACAGAAGAACATGGC-3′ and reverse: 5′-CCTGGTCATTCACCGTCTCA-3′), *PIP5K2C* (forward: 5′-CATCTTCCACTGCTAATGTGTCTCC-5′ and reverse: 5′-TTGAGTTATGGCTCTGACTCCTCTCT-3′), and β-actin (forward: 5′-GACGATATCGCTGCGCTGGT-3′ and reverse: 5′-CCACGATGGAGGGGAATA-3′), generating PCR products of 108, 104, 95, and 100 bp, respectively. Reactions were cycled 30 times (96°C for 1 minute, 60°C for 1 minute, 72°C for 3 minutes) on a Techne Progene thermal cycler and the products analyzed by 2% (w/v) agarose gel electrophoresis.

For in situ hybridization, two oligonucleotide probes for *PIP5K2C* were 3′-tail labeled with [^35^S]dATP (NEN, Hounslow, United Kingdom), hybridized with 20-μm mouse tissue sections, and autoradiographed for 5 weeks, as previously described (Giudici et al.,^2004^). Results for the probe designed to the 3′-untranslated region (5′-GACTGGGTGGATTGAGTTATGGCTCTGACTCCTCT-3′) were confirmed with the probe designed to the coding sequence (5′-ATAGGAGATAAGGAAACGGCCATCACTGCCTTCAG-3′). Both probes identified only *PIP5K2C* when BLAT searched (Kent,^2002^) against the EMBL mouse database. Slides were treated with autoradiographic emulsion and counterstained with methyl blue after development (12 weeks). Control incubations contained an excess of unlabeled probe in addition to the labeled oligonucleotide.

### Primary antibody characterization

PIP4Kγ was detected using a custom rabbit polyclonal antibody raised against a synthetic peptide representing amino acids 333–352 from the variable region of murine PIP4Kγ. This antibody specifically stains purified recombinant PIP4Kγ protein and a 47-kDa molecular weight band from mouse brain by Western blotting, and this signal is abolished in blotting and immunochemistry experiments by preincubation with excess antigenic peptide (this study; Clarke et al.,^2008^). Mouse monoclonal antibody to α-tubulin (Sigma-Aldrich, Gillingham, United Kingdom; catalog No. T9026, lot No. 093K4880) was raised against purified chick brain tubulin and stains a single band of 57-kDa molecular weight by Western blot (Blose et al.,^1984^). Mouse anti-calbindin D-28k monoclonal antibody [Swant, Bellinzona, Switzerland; code No. 300, lot No. 07(F)] was raised against purified chicken duodenum calbindin D-28k (Celio et al.,^1990^). The antibody recognizes a single band of 28 kDa by Western blot, and no specific immunohistochemical staining is seen in the brain of a calbindin D-28k knockout mouse (manufacturer's technical information). Antiparvalbumin mouse monoclonal antibody [Swant; code No. 235, lot No. 10-11(F)] was raised against purified carp muscle parvalbumin (Celio et al.,^1988^). The antibody specifically identifies 12-kDa, IEF-4.9 parvalbumin by 2D immunoblot and shows an absence of staining in the brain of a parvalbumin knockout mouse (manufacturer's technical information). Mouse monoclonal antibodies to GM130, early endosome antigen 1 (EEA1), and p115 (BD Transduction Laboratories, Oxford, United Kingdom; catalog Nos. 610822, 610456, and 612260) were raised to amino acids 869–982 of rat GM130, amino acids 3–281 of human EEA1, and amino acids 843–955 of rat p115, respectively. Each antibody stained a single band in rat brain by Western blot (130-kDa, 180-kDa, and 115-kDa molecular weights, respectively, by the manufacturer's technical information) and showed representative staining as previously reported (Allan et al.,^2000^; Clarke et al.,^2008^). The guinea pig polyclonal antibody against glial fibrillary acidic protein (GFAP; Advanced Immunochemical, Long Beach, CA; catalog No. 031223) was raised against purified human GFAP, and this antibody stained cells with the classic morphology and distribution of fibrous astrocytes in our experiments. Mouse monoclonal anti-neuronal class III β-tubulin (clone TUJ1; Stem Cell Technologies Inc., Vancouver, British Columbia, Canada; catalog No. 01409) was raised against rat brain microtubules and specifically stained neuronal and not glial cell β-tubulin (manufacturer's technical information). Mouse monoclonal anti-KDEL antibody (Stressgen, Ann Arbor, MI; catalog No. SPA-827) was raised against amino acids 649–654 of rat Grp78 (BiP) and recognized Grp78 (78 kDa) and Grp94 (94 kDa) bands in mouse brain by Western blot (manufacturer's technical information). Antibodies to golgin 160 (goat polyclonal), lysosome-associated membrane protein 1 (LAMP-1; mouse monoclonal), and LAMP-2 (rabbit polyclonal) were from Santa Cruz Biotechnology (Santa Cruz, CA; catalog Nos. sc-79966, sc-17768, and sc-5571, respectively). The antibody to golgin 160 was raised against the 14 N-terminal amino acids of human golgin 160 and stained cells with a pattern previously observed (Hicks and Machamer,^2002^). Anti-LAMP antibodies were raised against amino acids 1–228 and 1–207 of human LAMP-1 and LAMP-2, respectively, and stained single bands in cell lysates by Western blot (manufacturer's technical information) and cells with a pattern previously observed (Sarafian et al.,^1998^). Mouse monoclonal anti-Golgi 58K protein (Sigma; clone 58K-9; catalog No. G2404) was raised against rat liver Golgi 58K protein, stained a single 58-kDa band in rat brain, and gave a characteristic Golgi staining by immunofluorescence (Bloom and Brashear,^1989^). A mouse monoclonal anti-calnexin antibody and rabbit polyclonal antibodies to catalase, mannose-6-phosphate receptor, and mannosidase II were obtained from Abcam Ltd. (Cambridge, United Kingdom; catalog Nos. ab31290, ab1877, ab12894, and ab12277, respectively). Anti-calnexin was raised to calnexin of human origin and stained cells with a typical ER pattern (manufacturer's technical information). Anti-catalase antibody was raised against bovine liver catalase and gave a single band of 65 kDa by Western blot in a number of tissue lysates, as well as a characteristic peroxisomal staining pattern (manufacturer's technical information). Antibodies to mannose-6-phosphate receptor (amino acids 700–800) and mannosidase II were raised against protein of human origin and showed characteristic immunofluorescent staining patterns by the manufacturer's technical information.

### Western blotting

Whole mouse brains were dissected under a binocular microscope and separated into nine regions, and protein lysates were prepared in RIPA buffer (150 mM sodium chloride, 50 mM Tris pH7.4, 1 mM EDTA, 1% Triton X-100, 1% deoxycholic acid, 0.1% SDS) with 10 mM tetrasodium pyrophosphate, 10 mM sodium fluoride, 17.5 mM β-glycerophosphate, and 100 μl/ml protease inhibitor cocktail (Sigma-Aldrich; catalog No. P8340) as previously described (Clarke et al.,^2008^). Protein samples were resolved by 8–10% SDS-PAGE, and Western blots were carried out using antibodies to PIP4Kγ (1:1,000) or α-tubulin (1:5,000). Horseradish peroxidase-conjugated secondary antibody (anti-rabbit IgG or anti-mouse IgG, 1:5,000) and SuperSignal West Dura substrate (Pierce Protein Research Products, Rockford, IL) were used to detect positive signal via the manufacturer's protocol. Samples were dephosphorylated by treatment with 1 unit calf intestinal alkaline phosphatase (Promega, Madison, WI) per microgram protein for 60 minutes at 37°C. PIP4Kγ antibody for control blots was preincubated with excess antigenic peptide for 30 minutes at room temperature. Autoradiographic images were quantified by comparing average integrated pixel intensities (Image J; NIH, Bethesda, MD) for a defined area with background subtraction. These values were normalized to the loading control.

### Immunohistochemistry

Tissue was sectioned (20 μm) on a cryostat (Leica, Wetzlar, Germany) at –20°C and either mounted onto SuperFrost Plus Gold slides (Menzel-Glaser, Braunschweig, Germany) and stored at –80°C or stored as free-floating sections (0.1% sodium azide in PBS) at 4°C. Sagittal and horizontal brain sections for immunoperoxidase staining were pretreated for 20 minutes with 5% hydrogen peroxide in 20% methanol, blocked for 2 hours (1% fish skin gelatin, 0.3% Triton X-100 in PBS), and exposed to anti-PIP4Kγ antibody (1:500, 24 hours at 4°C). Detection was carried out with biotinylated anti-rabbit IgG secondary antibody (1:200), Vectastain ABC reagent, and DAB substrate (Vector Laboratories, Burlingame, CA). Free-floating stained sections were mounted onto gelatinized slides, air dried, and treated with ethanol (2 minutes in 70% solution, 2 minutes in 95% solution, 3 minutes in 100%) and then with Histoclear reagent (Sigma-Aldrich). Coverslips were permanently mounted with DPX reagent (Sigma-Aldrich).

Brain sections for fluorescent labeling experiments were pretreated and incubated as previously described (Clarke et al.,^2008^) using anti-PIP4Kγ (1:750), anti-calbindin D-28k (1:1,000), anti-parvalbumin (1:1,000), anti-GM130 (1:50), anti-p115 (1:100), anti-TUJ1 (1:1,000), anti-calnexin (1:1,000), and anti-GFAP (1:2,500). Fluorescent dye-conjugated secondary antibodies (Alexa Fluor 488 anti-rabbit IgG and Alexa Fluor 546 anti-rabbit, anti-mouse, and anti-guinea pig IgG, 1:1,000; Molecular Probes, Paisley, United Kingdom) were used for fluorescence microscopy. Whole sections for brain region reference were stained with 435/455 blue fluorescent Nissl stain (NeuroTrace; 1:300; Molecular Probes) with the manufacturer's specifications. Coverslips were mounted with ProLong Gold antifade reagent (Molecular Probes). PIP4Kγ antibody for controls was preincubated with 50 μg of recombinant protein for 30 minutes at room temperature. Brain areas were identified by using a stereotactic atlas (Lein et al.,^2007^).

### Immunocytochemistry

Primary hippocampal cultures were isolated and maintained as a source of pyramidal neurons (Koizumi et al.,^1999^), and primary cerebellar granule cells were isolated and maintained as described by Giudici et al. (2004). These cells were derived from rat brain, and PIP4Kγ from this source is 98.1% similar at the protein level and immunoreactive with the anti-PIP4Kγ antibody. Glass coverslips seeded with neuronal cultures or transiently transfected HeLa cells expressing GFP-tagged constructs were fixed in 4% PFA for 30 minutes on ice. Cells were permeabilized with 0.1% Triton X-100 in PBS for 10 minutes and blocked with 4% fish skin gelatin (Sigma-Aldrich) in PBS for 30 minutes, then incubated for 60 minutes with anti-PIP4Kγ (1:1,000), anti-GM130 (1:50), anti-p115 (1:100), anti-EEA1 (1:50), anti-mannose-6-phosphate receptor (1:500), anti-GRP78 (1:250), anti-58K (1:250), anti-mannosidase II (1:200), anti-golgin 160 (1:250), anti-catalase (1:200), anti-LAMP-1 (1:250), anti-LAMP-2 (1:250), or anti-tubulin (1:400). After incubation with secondary antibody (Alexa Fluor 488 anti-rabbit IgG and Alexa Fluor 546 anti-mouse or anti-goat IgG in 2% gelatin in PBS) or Alexa Fluor 568-conjugated phalloidin (Molecular Probes), coverslips were mounted onto slides with ProLong Gold antifade reagent (Molecular Probes) and analyzed as described below. For permeabilization experiments, cells were treated (prefixation) with 20 μg/ml digitonin or 1% Triton X-100 for 5 minutes (in 80 mM PIPES pH 6.8, 4% PEG 8000, 1 mM MgCl_2_, 1 mM EGTA).

### Microscopy and figure preparation

Light microscopy was performed with an Axioskop II microscope (Carl Zeiss GmbH, Munich, Germany), and images were saved as TIF format in AxioVision software (Carl Zeiss GmbH). Confocal microscopy was carried out by sequential scanning with a Leica TCS SP5 laser scanning confocal microscope running LAS AF software (Leica Microsystems Ltd., Milton Keynes, United Kingdom). Images were tinted and output as TIF files from Image J.

Autoradiographic images developed from in situ hybridization and electrophoresis gels were scanned and output as TIF files by using a Hewlett-Packard scanjet 8200 scanner and VueScan software (Hamrick Software, Pheonix, AZ). Figures were prepared for publication in Adobe Photoshop (Adobe Systems, San Jose, CA), adjusting image brightness and contrast only.

## RESULTS

### *PIP5K2C* has an expression profile in mouse brain

**Figure 1:**
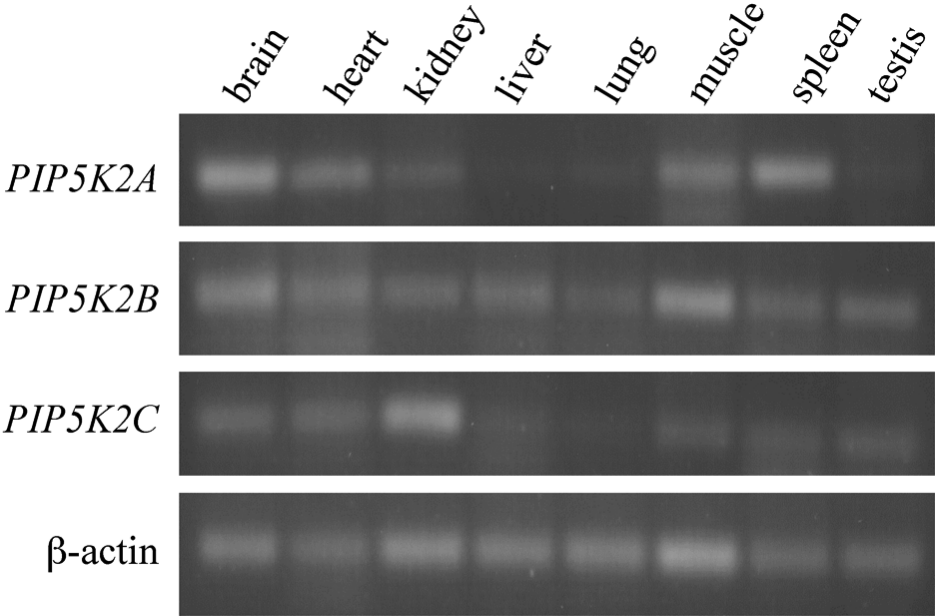
*PIP5K2* gene expression in adult mouse tissue cDNA libraries. Comparative levels of *PIP5K2A, PIP5K2B, PIP5K2C*, and β-actin control mRNA in different tissues as determined by RT-PCR (representative of results from cDNA libraries generated from three different animals).

**Figure 2:**
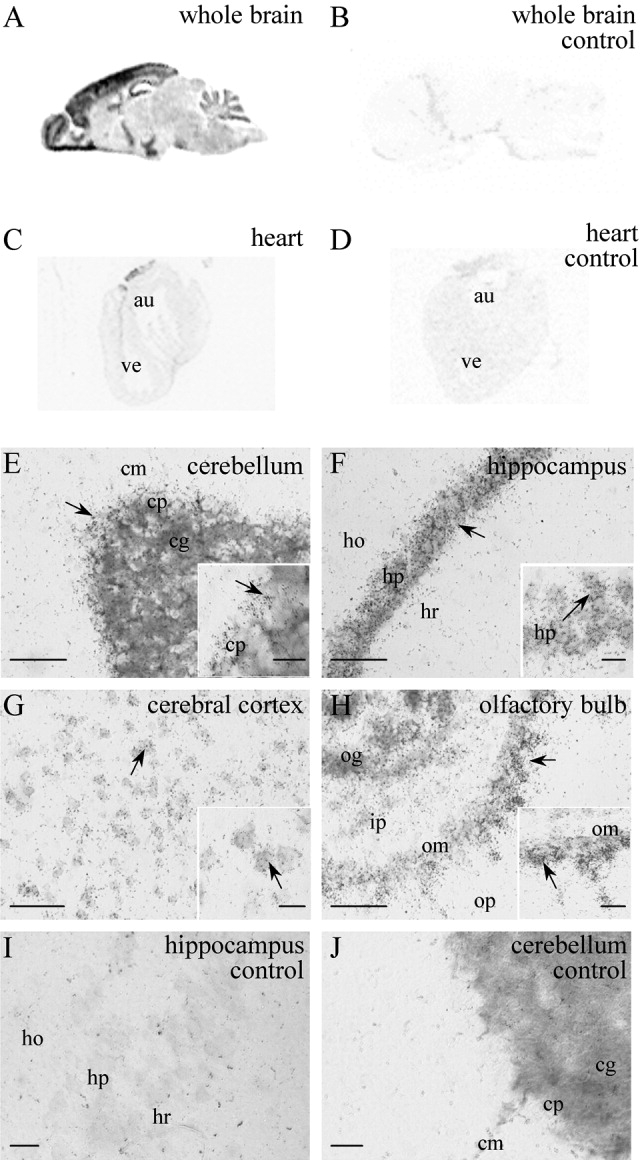
*PIP5K2C* gene expression in adult mouse tissues by in situ hybridization. With *PIP5K2C*-specific probes on 20-μm mouse tissue sections, positive signal is spatially distributed in brain (**A**) but absent from heart tissue (**C**; not to scale). Control sections of brain (**B**) and heart (**D**) were incubated with excess of unlabeled probe, and identical results were obtained with three sections from two animals. Autoradiographic emulsion staining of hybridization slides (counterstained with methyl blue), shows *PIP5K2C* mRNA labeled with silver grains (**E–J**). Positive cells (arrows) are seen in the cerebellum (E, **inset** is Purkinje cell layer), hippocampal field CA1 (F, **inset** is stratum pyramidale), cerebral cortex (G, **inset** is layer V), and olfactory bulb (H, **inset** is mitral cell layer). Controls incubated with excess cold probe (I,J) were negative. au, Heart auricle; ve, heart ventricle; cm, molecular layer; cp, Purkinje layer; cg, granular layer (all cerebellar); ho, stratum oriens; hp, stratum pyramidale; hr, stratum radiatum (all hippocampal); op, outer plexiform layer; om, mitral cell layer; ip, inner plexiform layer; og, granule cell layer (all olfactory bulb). Scale bars = 40 μm in E–I; 10 μm in J and insets.

Previous studies have suggested that *PIP5K2* isoforms are differentially transcribed according to tissue (Akiba et al.,^2002^; Clarke et al.,^2008^; Itoh et al.,^1998^), and RT-PCR with isoform-specific primers confirmed this (Fig. 1). Among the tissues tested, *PIP5K2A* mRNA levels were comparatively higher in mouse spleen, *PIP5K2B* levels were higher in muscle, and *PIP5K2C* was the predominant isoform in kidney (this study; Clarke et al.,^2008^). However, mRNA for each isoform was also detected, at different levels, in brain (Fig. 1). Tissue analysis by in situ hybridization with two different *PIP5K2C* probes confirmed that transcription of this isoform was up-regulated in discrete regions of the brain, compared with a control tissue (Fig. 2). Silver grain labeling of *PIP5K2C* mRNA in brain suggested that expression was confined to cells on the boundary of the molecular and granular layers of the cerebellum and in large cell bodies in the hippocampus (CA1–CA3), cerebral cortex, and olfactory bulb (Fig. 2).

### Endogenous PIP4Kγ is expressed in specific brain regions

**Figure 3:**
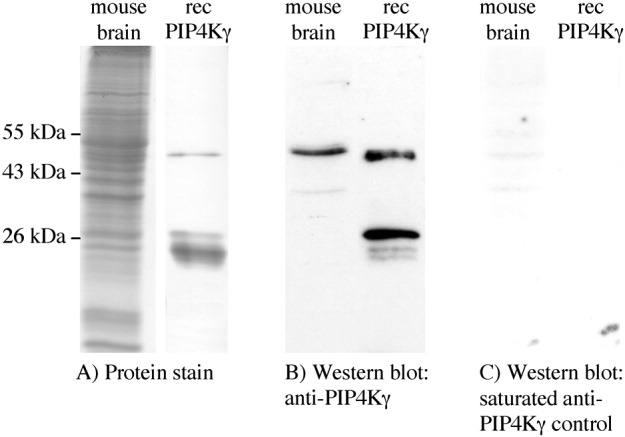
Specificity of antiphosphatidylinositol 5-phosphate 4-kinase γ (PIP4Kγ) antibody to PIP4Kγ in mouse brain. **A:** SDS-PAGE of total proteins from 50 μg whole mouse brain lysate (Coomassie stained) and 50 ng of purified recombinant (rec) PIP4Kγ (silver stained) showing mature protein of 47 kDa and degradation products at 26 kDa. **B:** Western blot of these proteins using anti-PIP4Kγ antibody, showing single band specificity in brain lysate. **C:** Control Western blot using anti-PIP4Kγ antibody preincubated with antigenic peptide. Results are representative of at least three experiments.

**Figure 4:**
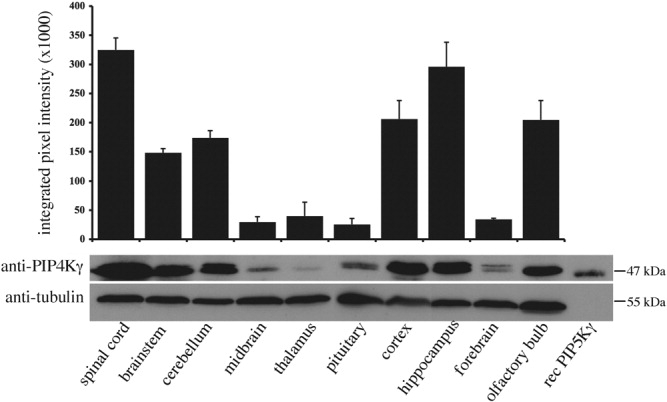
Endogenous PIP4Kγ expression in adult mouse brain. Quantification of PIP4Kγ levels in lysates (30 μg) from 10 different adult mouse CNS regions probed with PIP4Kγ-specific antibody (n = 3). Integrated pixel intensities for each band are shown (±SEM), normalized to the loading control (α-tubulin). Recombinant (rec) PIP4Kγ was included as a positive size control.

**Figure 5:**
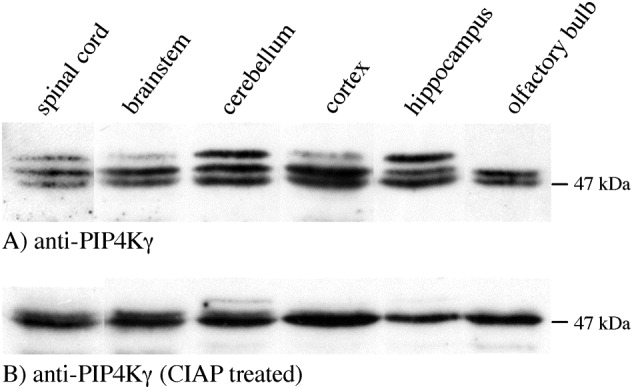
Multiple forms of PIP4Kγ are differentially expressed in brain regions. **A:** PIP4Kγ was detected as three different bands (47, 48, and 49 kDa) by 8% SDS-PAGE and Western blotting. The 49-kDa band was differentially expressed in cerebral cortex, hippocampus, spinal cord, cerebellum, and brainstem and was not present in the olfactory bulb. **B:** Only the 47-kDa band remained after lysates were pretreated with calf intestinal alkaline phosphatase (CIAP) and Western blotted with anti-PIP4Kγ antibody. Results are representative of at least three experiments.

With a polyclonal peptide antibody specific to the variable region of PIP4Kγ (Fig. 3; Clarke et al.,^2008^), our results indicated that this PIP4K isoform is differentially expressed within the mouse brain. Crude dissection of mouse brain tissue and Western blotting of the protein lysates allowed direct comparison of PIP4Kγ levels in these regions. Equivalent samples (by total protein loading) were standardized to the loading control (α-tubulin), and quantification indicated that PIP4Kγ levels were higher in the cerebral cortex, hippocampus, spinal cord, and olfactory bulb, with significant expression also seen in the cerebellum and brainstem (Fig. 4). Tissue lysates from these regions were further investigated to resolve multiple PIP4Kγ-immunoreactive bands. Western blotting of samples with comparative PIP4Kγ levels indicated the presence of three distinct bands at 47, 48, and 49 kDa (Fig. 5). The two lower molecular weight bands were present in all of the six regions, whereas the 49-kDa band was equivalent in the cerebellum and hippocampus; significantly reduced in the cortex, brainstem, and spinal cord; and completely absent from the olfactory bulb (Fig. 5). Treatment of samples with phosphatase removed the 48- and 49-kDa bands, suggesting that these were phosphorylated forms of PIP4Kγ (Fig. 5). Itoh et al. (1998) previously identified one phosphorylated form of PIP4Kγ, and our results suggest that the presence of a third immunoreactive band could indicate a form of the protein that is specific to distinct areas of brain tissue, in contrast to other tissues studied (Clarke et al.,^2008^).

**Figure 6:**
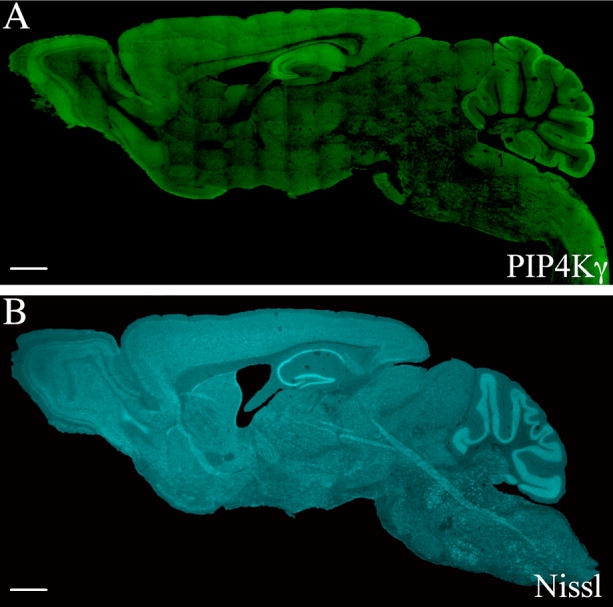
PIP4Kγ expression in different regions of the adult mouse brain. PIP4Kγ was detected in whole saggital brain sections (approximately lateral 0.48 mm) by immunofluorescence with anti-PIP4Kγ antibody (**A**). Staining indicated differential expression levels of PIP4Kγ in different brain regions. A similar, fluorescent Nissl-stained section is included for morphological reference (**B**). Images are representative of a minimum of six stained sections from three different mice. Scale bars = 1 mm. [Color figure can be viewed in the online issue, which is available at www.interscience.wiley.com.]

**Figure 7:**
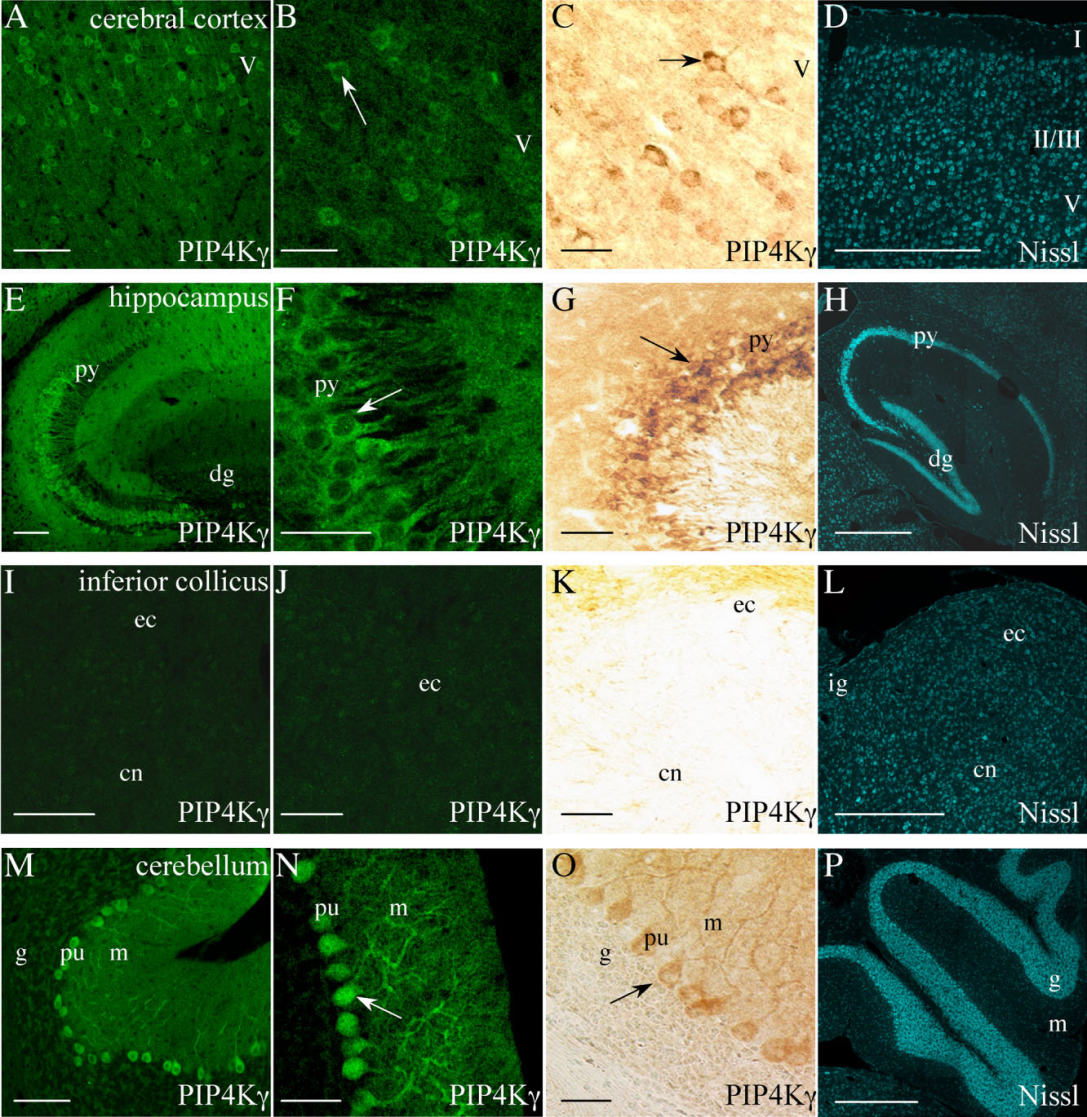
**A–P:** Localization of PIP4Kγ in adult mouse brain regions. Positive PIP4Kγ signal (arrows) was observed in the cerebral cortex (A–C), CA1–CA3 of the hippocampus (E–G) and cerebellum (M–O). No significant signal was observed in the inferior colliculus (I–K). Saggital brain sections were stained by immunofluorescent (A,B,E,F,I,J,M,N) and also immunochemical (C,G,K,O) methods. Fluorescent Nissl-stained images are included for reference (D,H,L,P). Images are representative of a minimum of six stained sections from three different mice. I, II/III, V, cerebral cortex layers; dg, dentate gyrus; py, hippocampal pyramidal cells; ec, external cortex of the inferior collicus; cn, central nucleus of the inferior collicus; ig, intermediate gray layer of the superior collicus; pu, cerebellar Purkinje cells; g, cerebellar granular layer; m, cerebellar molecular layer. Scale bars = 100 μm in A,E,I,M; 50 μm in B,F,J,N; 20 μm in C,G,K,O; 500 μm in D,H,L,P. [Color figure can be viewed in the online issue, which is available at www.interscience.wiley.com.]

**Figure 8:**
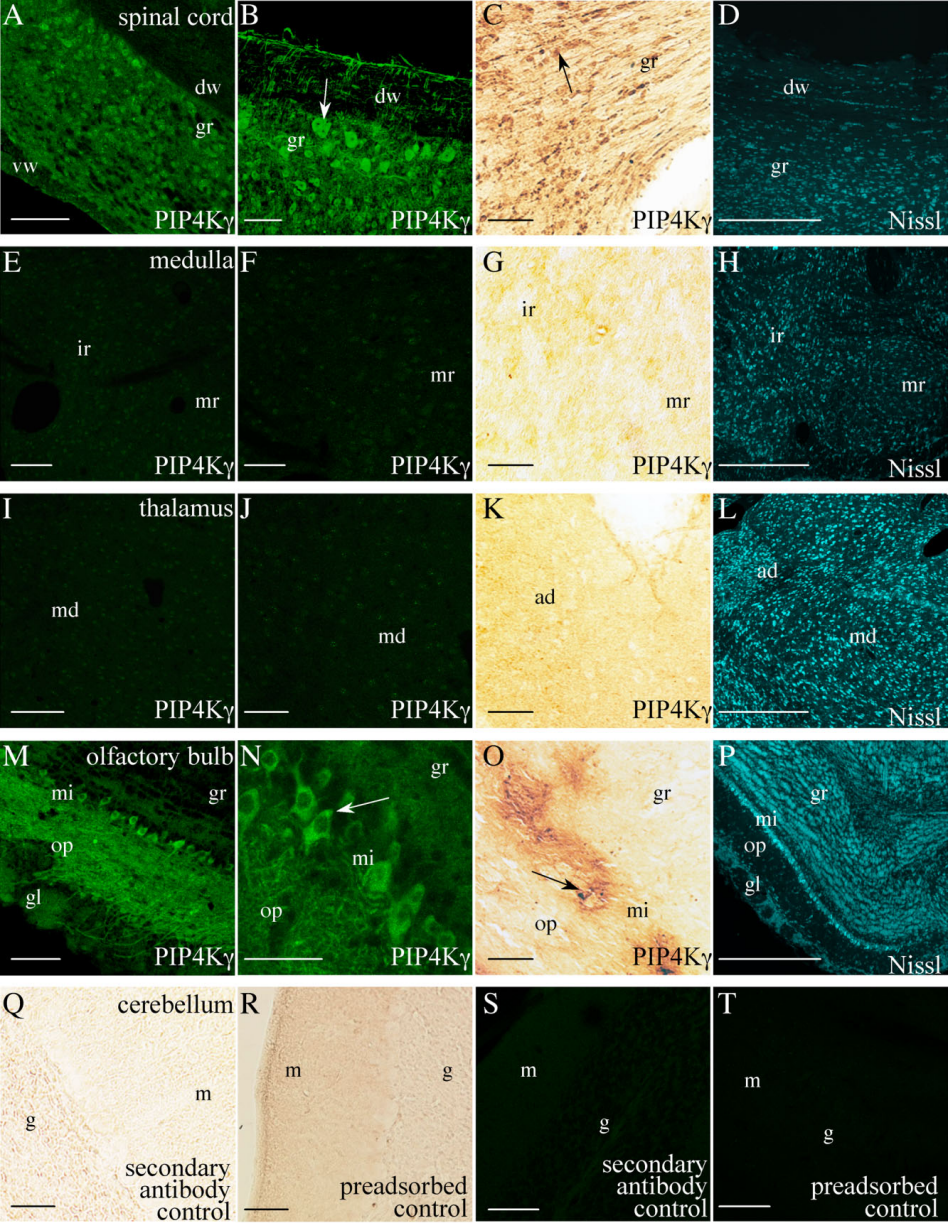
**A–T:** Localization of PIP4Kγ in adult mouse brain regions. Positive PIP4Kγ signal (arrows) was observed in the cervical spinal cord (longitudinal section, A–C) and olfactory bulb (M–O). No significant signal was observed in other areas such as the medulla (E–G) or thalamus (I–K). Saggital brain sections were stained by immunofluorescent (A,B,E,F,I,J,M,N,S,T) and also immunochemical (C,G,K,O,Q,R) methods. Fluorescent Nissl-stained images are included for reference (D,H,L,P). Controls for immunochemical and immunofluorescent labeling (incubated with secondary antibody only or using preadsorbed anti-PIP4Kγ) are also included (Q–T). Images are representative of a minimum of six stained sections from three different mice. dw, Dorsal white matter of the spine; vw, ventral white matter of the spine; gr, spinal gray matter; ir, intermediate reticular nucleus of the medulla; mr, medullary reticular nucleus; md, mediodorsal/paracentral thalamic nuclei; ad, anterodorsal thalamic nucleus; gl, olfactory glomerular layer; op, olfactory outer plexiform layer; gr, olfactory granule cell layer; mi, olfactory mitral cells; g, cerebellar granular layer; m, cerebellar molecular layer. Scale bars = 100 μm in A,E,I,M; 50 μm in B,F,J,N; 20 μm in C,G,K,O,Q–T; 500 μm in D,H,L,P. [Color figure can be viewed in the online issue, which is available at www.interscience.wiley.com.]

Immunostaining of endogenous PIP4Kγ in whole-brain sections allowed the further identification of regions showing localized expression of PIP4Kγ (Fig. 6). Detailed examination of sections, labeled with anti-PIP4Kγ by immunochemical or immunofluorescent methods, indicated expression of this isoform in isolated cells (including pyramidal and basket cells) throughout the cerebral cortical layers (Fig. 7A–C), and this expression did not extend into the corpus callosum or cingulum region or into the caudate-putamen. High levels of expression were seen in the hippocampal formation, primarily in the stratum pyramidale and extending into the stratum radiatum of CA1–CA3, and were excluded from the dentate gyrus (Fig. 7E–G). Within the cerebellar cortex, PIP4Kγ was observed in large cell bodies of the Purkinje layer, extending into the dendritic trees of these cells, but was absent from the granular layer (Fig. 7M–O). PIP4Kγ expression was excluded from the majority of the medulla (Fig. 8E–G) but was present in the dorsal horn of the spinal cord (Fig. 8A–C) and the spinal trigeminal nucleus and tract (data not shown). Positive signal was also observed throughout the cervical, thoracic, and lumber spinal cord regions (data not shown) and large neuronal cell bodies in the dorsal root ganglia (see Fig. 12C). Lower level expression was observed in the pontine gray, and the remainder of the pons, midbrain, thalamus, and hypothalamus were negative (Figs. 7I–K, 8I–K). In the olfactory bulb, PIP4Kγ expression was seen in cells extending from the mitral cell layer to the olfactory nerve layer and into the anterior olfactory nucleus but excluded from the granular cell layer (Fig. 8M–O).

### Expression of PIP4Kγ is restricted to specific neurons

**Figure 9:**
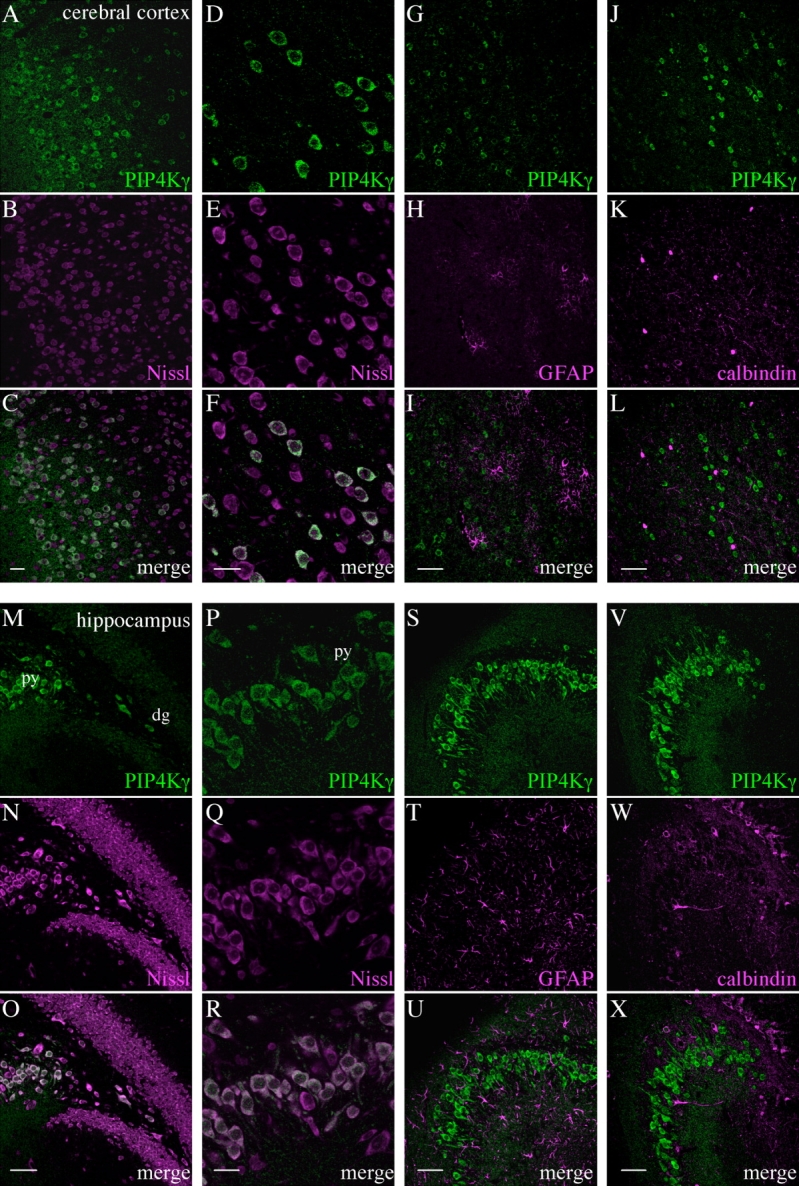
Identification of cell types expressing PIP4Kγ in the adult mouse cerebral cortex and hippocampus. Endogenous PIP4Kγ was detected in cerebral cortex (**A–L**) and hippocampus (**M–X**) by using anti-PIP4Kγ antibody (green). Counterstaining for Nissl substance (B,E,N,Q) indicated that PIP4Kγ was expressed in neuronal cells (C,F,O,R). PIP4Kγ was excluded from glial cells stained with anti-glial fibrillary acidic protein (GFAP; G–I,S–U). PIP4Kγ was not expressed in cells that were positive for the neuronal cell marker calbindin D-28k in these regions (J–L,V–X). Images are representative of three stained sections. py, Stratum pyramidale of hippocampus; dg, dentate gyrus. Scale bars = 50 μm in C (applies to A–C); 20 μm in F (applies to D–F); 50 μm in I (applies to G–I); 50 μm in L (applies to J–L); 50 μm in O (applies to M–O); 20 μm in R (applies to P–R); 50 μm in U (applies to S–U); 50 μm in X (applies to V–X).

**Figure 10:**
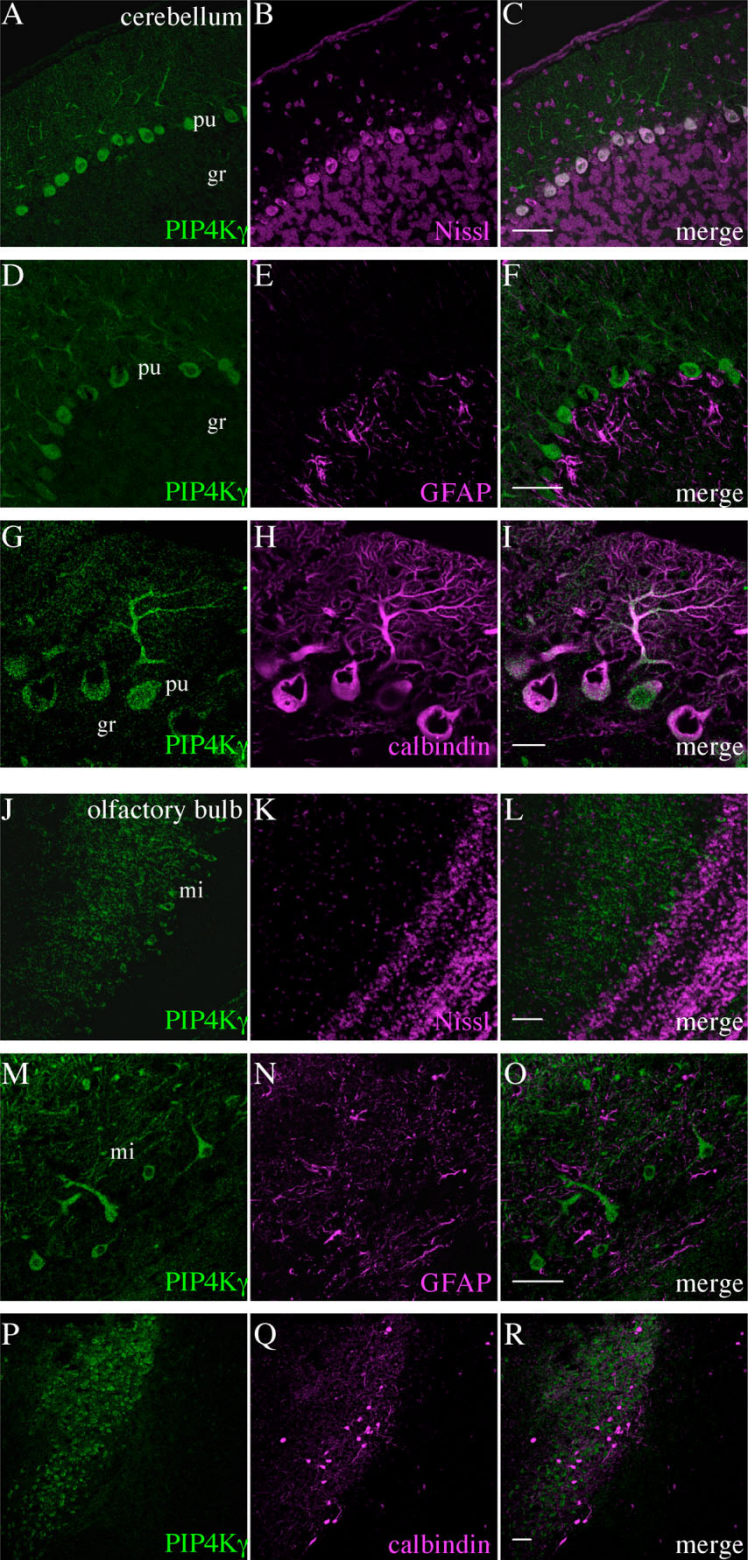
Identification of cell types expressing PIP4Kγ in the adult mouse cerebellum and olfactory bulb. PIP4Kγ (green) was detected in the cerebellum (**A–I**) and olfactory bulb (**J–R**). Signal was coincident with fluorescent Nissl stain (A–C,J–L) and was excluded from glial cells stained with anti-GFAP (D–F,M–O), indicating that PIP4Kγ was expressed in neuronal cells. PIP4Kγ was expressed in cells that were positive for the neuronal cell marker calbindin D-28k in the cerebellum (G–I) but not in the olfactory bulb (P–R). Images are representative of three stained sections. gr, Cerebellar granular layer; pu, cerebellar Purkinje layer; mi, mitral cell layer of olfactory bulb. Scale bars = 50 μm in C (applies to A–C); 50 μm in F (applies to D–F); 20 μm in I (applies to G–I); 50 μm in L (applies to J–L); 20 μm in O (applies to M–O); 50 μm in R (applies to P–R).

**Figure 11:**
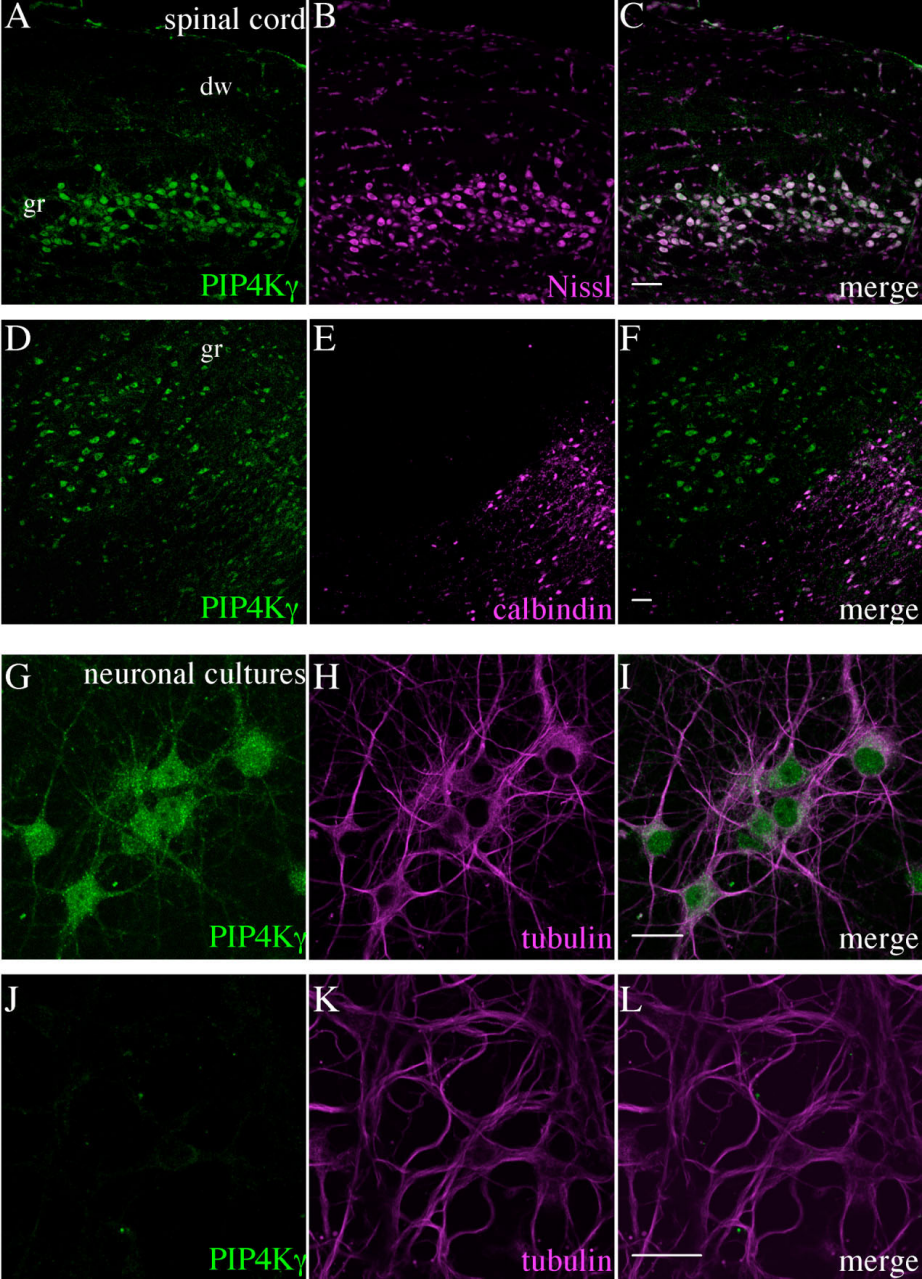
Identification of cell types expressing PIP4Kγ in the adult mouse spinal cord and in primary neuronal cultures. Endogenous PIP4Kγ (green) was detected in neurons in the spinal cord (**A–F**) by fluorescent costaining for Nissl substance (A–C) and was also seen in the subpopulation of neurons positive for calbindin D-28k (D–F). Hippocampal primary cell cultures enriched for pyramidal cells expressed PIP4Kγ (**G–I**), whereas cultures enriched for cerebellar granule cells did not (**J–L**). Images for (A–F) are representative of three stained sections. dw, Dorsal white matter of the spine; gr, spinal gray matter. Scale bars = 50 μm in C (applies to A–C); 50 μm in F (applies to D–F); 20 μm in I (applies to G–I); 20 μm in L (applies to J–L).

Further characterization of PIP4Kγ-positive cells in the adult mouse brain indicated that this phosphoinositide kinase is preferentially expressed in cells that are costained with neuronal cell markers (Figs. 9–11; Supp. Info. Figs. 1, 2). Use of a fluorescent counterstain to identify neurons by abundance of Nissl substance (Quinn et al.,^1995^) colocalized PIP4Kγ to these cells in each region studied (Figs. 9C,F,O,R, 10C,L, 11C), and this was confirmed by colocalization with the neuronal class III β-tubulin marker TUJ1 (Supp Info. Fig. 1). PIP4Kγ was not expressed in granule cells in the cerebellum (Fig. 10C) or in the dentate gyrus (Fig. 9O). Costaining with an antibody to the glial cell marker GFAP (Figs. 9I,U, 10F,O) suggested that the PIP4Kγ signal was excluded from these cells. Furthermore, staining of pyramidal cell cultures (derived from hippocampus) for endogenous PIP4Kγ indicated that these neurons were PIP4Kγ positive (Fig. 11I), whereas primary cell cultures of granule cells (derived from cerebellum) showed little endogenous signal (Fig. 11L). Within the cerebral cortex, hippocampus, and olfactory bulb, the subset of neurons expressing PIP4Kγ was excluded from the subpopulations expressing the calcium-binding protein calbindin D-28k (Figs. 9L,X, 10R), and parvalbumin (Supp. Info. Fig. 2C,F,L), a calcium-binding marker of GABAergic interneurons, which is predominantly expressed by chandelier and basket cells in the cortex (Hendry et al.,^1989^). In the cerebellum, both of these markers were present in PIP4Kγ-positive Purkinje cells (Fig. 10I; Supp Info. Fig. 2I), and, within the spinal cord, the population of PIP4Kγ-positive neurons included subpopulations positive for both calbindin D-28k (Fig. 11F) and parvalbumin (Supp. Info. Fig. 2O).

### PIP4Kγ has a distinct subcellular compartmentalization

**Figure 12:**
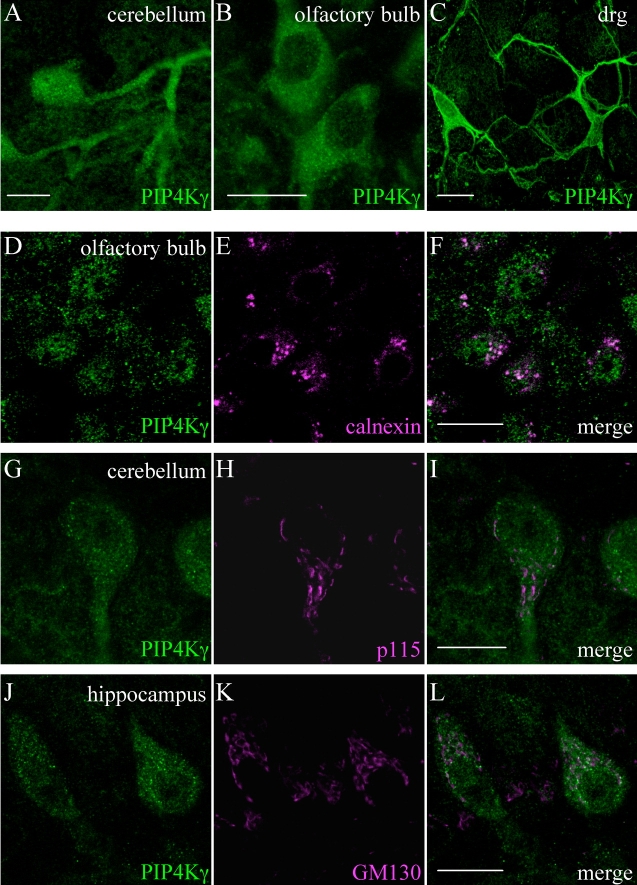
Subcellular localization of PIP4Kγ in neurons. Endogenous PIP4Kγ was detected in the cell body and dendritic trees of cerebellar Purkinje cells (**A**), olfactory bulb mitral cells (**B**), and large neurons in dorsal root ganglion (drg) preparations (**C**). PIP4Kγ was mostly excluded from the nucleus and was present in a vesicular compartment in the cytoplasm. This compartment seemed to have a stronger, but partial, colocalization with the *cis*-Golgi structural marker GM130 (**J–L**) than with the ER resident calnexin (**D–F**) or the -ergic marker p115 (**G–I**), although this could not be fully characterized in brain tissue. Images are representative of results from at least three stained sections. Scale bars = 20 μm in A–C; 20 μm in F (applies to D–F); 20 μm in I (applies to G–I); 20 μm in L (applies to J–L).

Confocal microscopy at high resolution and costaining with a range of markers for different subcellular compartments allowed the identification of a distinct vesicular location for PIP4Kγ. In all of the neurons observed expressing PIP4Kγ, endogenous signal was present in the cell body and dendritic projections but was mostly excluded from the nucleus (Fig. 12). Subcellular localization was cytoplasmic and partially perinuclear, with clear, punctate staining (Fig. 12A–C), similar to that observed in kidney cells (Clarke et al.,^2008^). Costaining of endogenous PIP4Kγ in brain tissue with markers for the endoplasmic reticulum and Golgi apparatus suggested that the vesicular PIP4Kγ compartment might partially colocalize with one of these structures (Fig. 12F,I,L).

**Figure 13:**
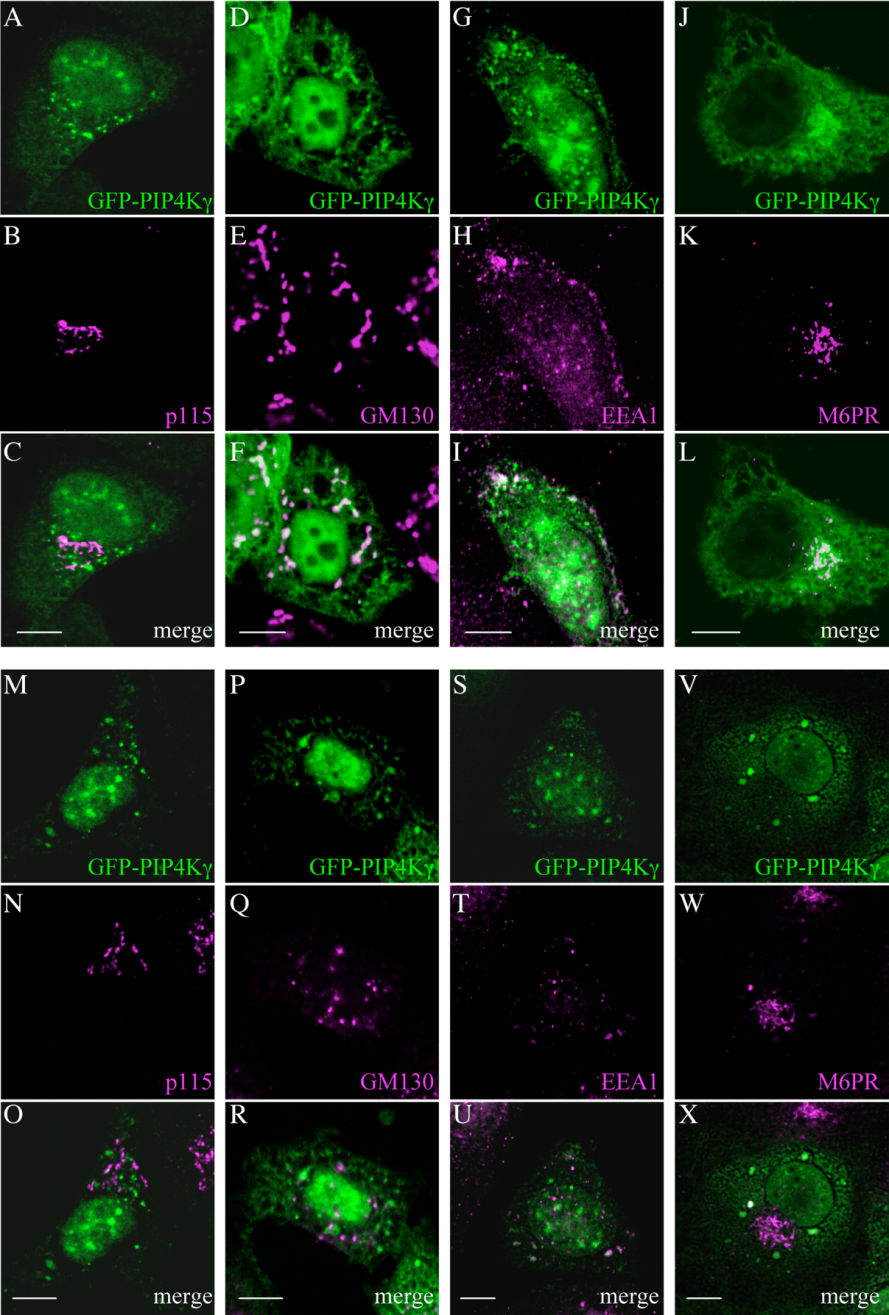
PIP4Kγ associates with a vesicular compartment that partially colocalizes with components of the endomembrane system. Expression of GFP-tagged PIP4Kγ in HeLa cells and mild permeabilization with digitonin (**A–L**) indicated a vesicular cytoplasmic pool of PIP4Kγ that partially colocalized with the Golgi marker GM130 (D–F) and the endosomal markers EEA1 (G–I) and mannose-6-phosphate receptor (M6PR; J–L) but not with the -ergic marker p115 (A–C). Results observed after permeabilization with Triton X-100 (**M–X**) suggested that the main pool of PIP4Kγ did not remain localized to any of these markers (O,R,U,X). Scale bars = 10 μm in C (applies to A–C); 10 μm in F (applies to D–F); 10 μm in I (applies to G–I); 10 μm in L (applies to J–L); 10 μm in O (applies to M–O); 10 μm in R (applies to P–R); 10 μm in U (applies to S–U); 10 μm in X (applies to V–X).

To investigate this potential association in more detail, HeLa cells were transiently transfected with constructs expressing PIP4Kγ fused to the GFP reporter protein, because very low endogenous levels of PIP4Kγ were observed in neuronal cell lines such as SH-SY5Y (data not shown). Cells were permeabilized to reduce the levels of free cytosolic protein produced by overexpression, as judged by the reduction of overexpressed control GFP levels (Supp. Info. Fig. 3). Treatment with the glycoside digitonin (Lorenz et al.,^2006^) significantly reduced cytosolic levels of GFP and all three PIP4K isoforms, and stronger detergent extraction completely removed GFP signal, but nuclear PIP4Kβ and vesicular PIP4Kγ remained resistant to this procedure, suggesting a stronger association with a cellular compartment (Supp. Info. Fig. 3). A proportion of PIP4Kγ remains localized to the nucleus during extraction, which may also be significant. Permeabilization did not affect the staining of other cellular compartment markers. PIP4Kγ was not seen to colocalize with the endoplasmic reticulum resident Grp78 (Supp. Info. Fig. 5C), in accordance with previous observations (Clarke et al.,^2008^), or the -ergic Golgi vesicle docking protein p115 (Fig. 13C). Partial colocalization after digitonin treatment was observed with the Golgi markers GM130 (Fig. 13F), 58K, mannosidase II, and golgin 160 (Supp. Info. Fig. 4) and the endosomal markers EEA1 and mannose-6-phosphate receptor (Fig. 13I,L), but not the lysosomal markers LAMP-1 and LAMP-2 or the peroxisomal marker catalase (Supp. Info. Fig. 5F,I,L). However, the PIP4Kγ protein remaining after stronger detergent treatment did not completely localize to any of these compartments (Fig. 13O,R,U,X; Supp. Info. Fig. 6).

### PIP4Kγ expression during postnatal development

**Figure 14:**
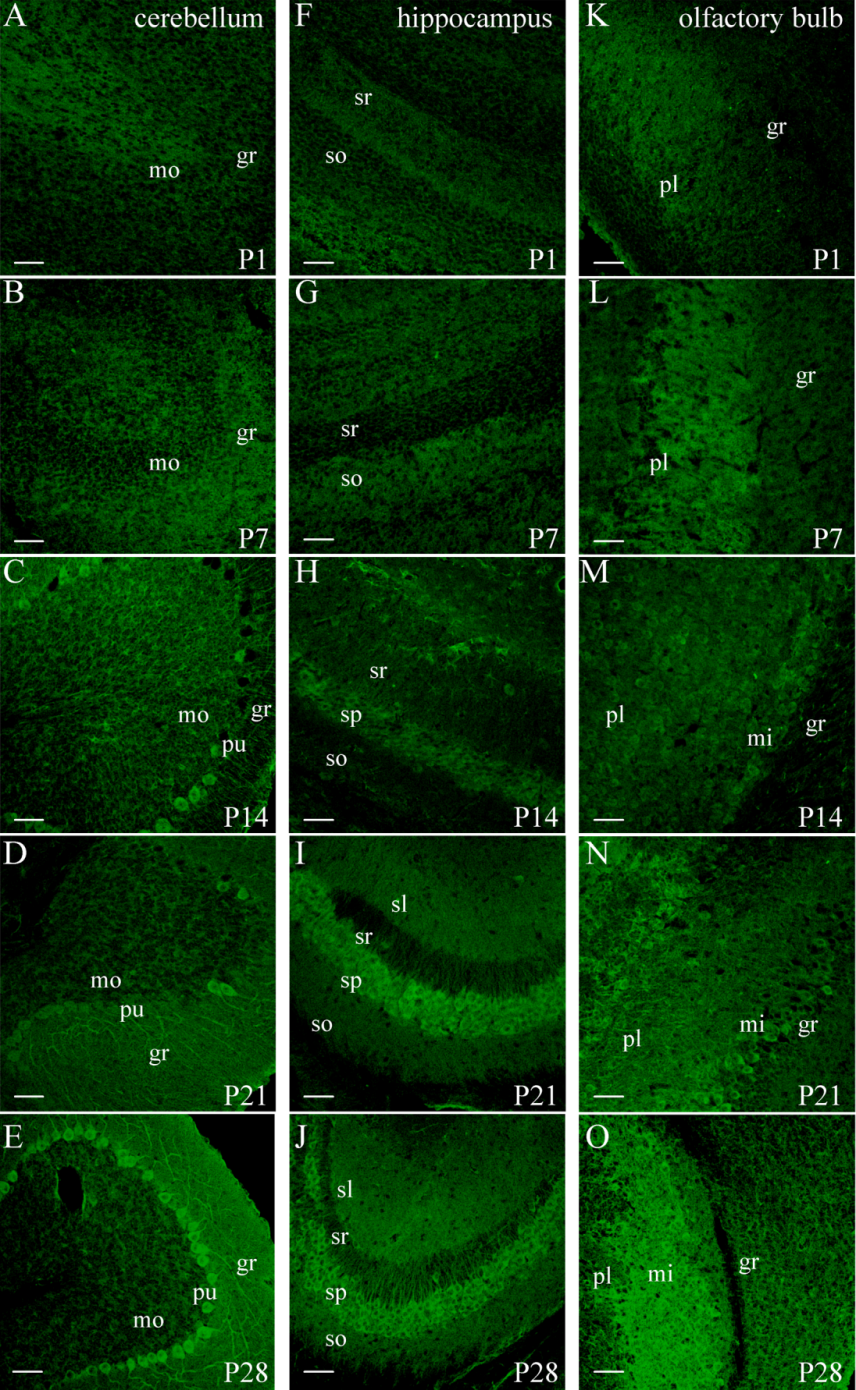
Expression of PIP4Kγ in the developing mouse brain. Immunohistochemical staining of mouse brain sections at different postnatal development stages (P1–P28 days after birth) indicated the levels of PIP4Kγ (green) detected in three different regions; cerebellum (**A–E**), hippocampal field CA3 (**F–J**), and olfactory bulb (**K–O**). Brain regions are pictured in the same orientation, and images are representative of two animals at each developmental stage. mo, Molecular layer; pu, Purkinje layer; gr, granular layer (cerebellum); so, stratum oriens; sp, stratum pyramidale; sr, stratum radiatum; sl, stratum lacunosum-moleculare (hippocampal formation); pl, plexiform layer; mi, mitral cell layer; gr, granule layer (olfactory bulb). Scale bars = 50 μm. [Color figure can be viewed in the online issue, which is available at www.interscience.wiley.com.]

Examination of similar brain sections in a murine postnatal developmental series indicated that the onset of PIP4Kγ expression from neurons was between postnatal days 7 and 14 (Fig. 14). Establishment of Purkinje cells and defined dendritic trees in the Purkinje layer of the cerebellum was apparent at P14, and staining for endogenous PIP4Kγ indicated that this enzyme was also expressed by Purkinje cells at this time. Spaces in the Purkinje cell layer may be due to naturally occurring neuronal cell death (Madalosso et al.,^2005^). Similar results were observed for PIP4Kγ expression in hippocampal pyramidal cells and the mitral cell layer of the olfactory bulb (Fig. 14).

## DISCUSSION

Here we have investigated the expression of the PIP4K isoforms in the murine brain, with specific reference to PIP4Kγ. We have further characterized the restricted localization of this PIP4K in different regions of the brain, identified the neuronal cells expressing the protein, and suggested a subcellular compartmentalization that has implications for the physiological function of this isoform.

### PIP4K expression and neuronal localization

Gene expression profiles for the *PIP5K2*s, collated as MIAME-compliant microarray data on the EMBL ArrayExpress Warehouse database (Parkinson et al.,^2007^), support the observations that there are tissue-specific differences in isoform abundance (Clarke et al.,^2008^). PIP4Kα is the most abundant isoform in spleen and blood components, such as platelets (Hinchliffe et al.,^1998^; Morris et al.,^2000^), whereas PIP4Kβ levels are enhanced in skeletal muscle (Castellino et al.,^1997^), and PIP4Kγ is most abundant in kidney (Clarke et al.,^2008^; Itoh et al.,^1998^). However, all three PIP4K isoforms are also transcribed in specific areas of the mammalian brain but have different spatial distributions (Akiba et al.,^2002^), and our in situ hybridization studies conform to the *PIP5K2C* gene expression pattern reported in the Allen Brain Atlas (Lein et al.,^2007^). Our detection of endogenous PIP4Kγ protein in brain is consistent with these transcription levels, and the results from Western blotting of nine different brain regions concur with the results obtained from immunohistochemistry on brain sections, with the majority of protein expression observed in the cerebral cortex, hippocampus, cerebellum, and olfactory bulb. We also note a significant level of expression in the spinal cord, and specifically in the large neuronal bodies found in the dorsal root ganglia. Within the brain regions, PIP4Kγ was expressed in specific neuronal cell populations, and expression in granule cells and neuroglia was negligible. These neurons could be identified as Purkinje cells in the cerebellum, pyramidal cells in the hippocampus, and cells in the mitral cell layer of the olfactory bulb. The expression of PIP4Kγ was also seen to be homogeneous in these areas and not restricted to neuronal populations within specific hippocampal subfields or cerebellar compartments. The population of PIP4Kγ-positive neuronal cells in the cerebral cortex and spinal cord was a defined subset, based on our marker studies, and requires further characterization.

### PIP4Kγ and neuronal development

Gene ontology (GO) terms (Ashburner et al.,^2000^) for neuron-enriched gene expression in the CNS suggest that there are categories involved with synaptic function, neuronal cell signalling and metabolism, neurogenesis, and neuronal transport, including vesicle-mediated trafficking (Lein et al.,^2007^). Recent reports have also implicated PtdIns5P (Xie et al.,^2005^) and PI-metabolizing enzymes (Coulson et al.,^2008^; Polleux et al.,^2002^) in neurogenesis and neuronal development and migration. Our results indicate that, in the developing postnatal mouse brain, PIP4Kγ expression is first observed after P7 and is established at P14, a developmental stage at which neurogenesis and neuronal migration and differentiation of Purkinje cells in the cerebellum, pyramidal cells in the hippocampus, and mitral cells in the olfactory bulb should be completed (Finlay and Darlington,^1995^; Hatten,^1999^; Madalosso et al.,^2005^). The lack of observed PIP4Kγ expression before postnatal stage P7 would suggest that this PIP4K does not participate in phosphoinositide-regulated events prior to this, but this has yet to be determined for embryonic developmental stages.

### PIP4Kγ subcellular distribution and neuronal function

The expression of PIP4Kγ in a neuronal subpopulation from P14 to adult would suggest that this enzyme might have a specific involvement with neuronal function. Our observation that PIP4Kγ is present in large neuronal cell bodies, as well as throughout their dendritic projections, would suggest a central rather than a peripheral role. Our results also suggest that, as with specialized cells in the mammalian nephron (Clarke et al.,^2008^), PIP4Kγ is localized to a vesicular cytoplasmic compartment that is partially coincident with components of the endomembrane system. The absence of a recognized signal peptide within the PIP4Kγ sequence suggests that this protein would not be directly targeted to the ER or plasma membrane, in accordance with our colocalization studies, and hence may be associated with the external surface of a trafficking compartment (Alvarez et al.,^2001^). Our evidence of partial colocalization of PIP4Kγ with Golgi complex and endosomal markers may suggest that this protein is associated with specific transport vesicles that shuttle between different endosomal organelles. The presence of PtdIns5P in trafficking vesicles is suggested by the substrate preference of a Golgi-localized lipid phosphatase, PLIP (Merlot et al.,^2003^), and the involvement of this phosphoinositide with vesicle translocation to the plasma membrane (Sbrissa et al.,^2004^). Furthermore, a recent comparative genomics study suggests that PtdIns5P is regulated by protein complexes, involving a kinase such as PIP4K, and has a role in membrane trafficking (Lecompte et al.,^2008^). PIP4Kγ has also been associated with actin remodelling during endocytic transport (Pelkmans et al.,^2005^), suggesting that modification of the PtdIns5P signal or synthesis of PtdIns(4,5)P_2_ in a specific compartment might have a role in this process. Recombinant PIP4Kγ has no detectable intrinsic kinase activity, although enzyme recovered from a mammalian expression system is seen to be active (Itoh et al.,^1998^). Our previous study suggested that this activity could also be attributable to the ability of PIP4Kγ to associate with other, more active PIP4K isoforms (Clarke et al.,^2008^) and that PIP4Kγ might function to recruit this activity to a specific cellular compartment. It should also be noted that there have been several recent reports of the regulation of various membrane channels by phosphoinositides (for reviews see Gamper and Shapiro,^2007^; Suh and Hille,^2005^), which could be coincident with delivery of the channel complexes to the plasma membrane. Although there is as yet no direct evidence for the involvement of PIP4Kγ in these processes, it remains an intriguing possibility that the PIP4Ks are involved in neuronal function via one of these mechanisms.

The restricted expression of PIP4Kγ in the CNS may be significant within the context of the specialized function of different neurons. It remains to be seen whether the role of phosphoinositide signalling involving this kinase is a common feature to the population of neurons that express it or whether there are different, neuron-specific functions of PtdIns5P, or localized pools of PtdIns(4,5)P_2_, in these cells.

## References

[b1] Akiba Y, Suzuki R, Saito-Saino S, Owada Y, Sakagami H, Watanabe M, Kondo H (2002). Localization of mRNAs for phosphatidylinositol phosphate kinases in the mouse brain during development. Brain Res Gene Expr Pattern.

[b2] Allan BB, Moyer BD, Balch WE (2000). Rab1 recruitment of p115 into a cis-SNARE complex: programming budding COPII vesicles for fusion. Science.

[b3] Alvarez C, Garcia-Mata R, Hauri HP, Sztul E (2001). The p115-interactive proteins GM130 and giantin participate in endoplasmic reticulum-Golgi traffic. J Biol Chem.

[b4] Ashburner M, Ball CA, Blake JA, Botstein D, Butler H, Cherry JM, Davis AP, Dolinski K, Dwight SS, Eppig JT, Harris MA, Hill DP, Issel-Tarver L, Kasarskis A, Lewis S, Matese JC, Richardson JE, Ringwald M, Rubin GM, Sherlock G (2000). Gene ontology: tool for the unification of biology. The Gene Ontology Consortium. Nat Genet.

[b5] Bloom GS, Brashear TA (1989). A novel 58-kDa protein associates with the Golgi apparatus and microtubules. J Biol Chem.

[b6] Blose SH, Meltzer DI, Feramisco JR (1984). 10-nm filaments are induced to collapse in living cells microinjected with monoclonal and polyclonal antibodies against tubulin. J Cell Biol.

[b7] Bunce MW, Boronenkov IV, Anderson RA (2008). Coordinated activation of the nuclear ubiquitin ligase Cul3-SPOP by the generation of phosphatidylinositol 5-phosphate. J Biol Chem.

[b8] Castellino AM, Parker GJ, Boronenkov IV, Anderson RA, Chao MV (1997). A novel interaction between the juxtamembrane region of the p55 tumor necrosis factor receptor and phosphatidylinositol-4-phosphate 5-kinase. J Biol Chem.

[b9] Celio MR, Baier W, Scharer L, de Viragh PA, Gerday C (1988). Monoclonal antibodies directed against the calcium binding protein parvalbumin. Cell Calcium.

[b10] Celio MR, Baier W, Scharer L, Gregersen HJ, de Viragh PA, Norman AW (1990). Monoclonal antibodies directed against the calcium binding protein calbindin D-28k. Cell Calcium.

[b11] Ciruela A, Hinchliffe KA, Divecha N, Irvine RF (2000). Nuclear targeting of the beta isoform of type II phosphatidylinositol phosphate kinase (phosphatidylinositol 5-phosphate 4-kinase) by its alpha-helix 7. Biochem J.

[b12] Clarke JH, Emson PC, Irvine RF (2008). Localization of Phosphatidylinositol phosphate kinase IIγ in kidney to a membrane trafficking compartment within specialized cells of the nephron. Am J Physiol Renal Physiol.

[b13] Coulson EJ, May LM, Osborne SL, Reid K, Underwood CK, Meunier FA, Bartlett PF, Sah P (2008). p75 Neurotrophin receptor mediates neuronal cell death by activating GIRK channels through phosphatidylinositol 4,5-bisphosphate. J Neurosci.

[b14] Di Paolo G, De Camilli P (2006). Phosphoinositides in cell regulation and membrane dynamics. Nature.

[b15] Di Paolo G, Moskowitz HS, Gipson K, Wenk MR, Voronov S, Obayashi M, Flavell R, Fitzsimonds RM, Ryan TA, De Camilli P (2004). Impaired PtdIns(4,5)P_2_ synthesis in nerve terminals produces defects in synaptic vesicle trafficking. Nature.

[b16] Endersby R, Baker SJ (2008). PTEN signaling in braneuropathology and tumorigenesis. Oncogene.

[b17] Fedorenko O, Strutz-Seebohm N, Henrion U, Ureche ON, Lang F, Seebohm G, Lang UE (2008). A schizophrenia-linked mutation in PIP5K2A fails to activate neuronal M channels. Psychopharmacology.

[b18] Finlay BL, Darlington RB (1995). Linked regularities in the development and evolution of mammalian brains. Science.

[b19] Gamper N, Shapiro MS (2007). Regulation of ion transport proteins by membrane phosphoinositides. Nat Rev Neurosci.

[b20] Giudici ML, Emson PC, Irvine RF (2004). A novel neuronal-specific splice variant of type I phosphatidylinositol 4-phosphate 5-kinase isoform gamma. Biochem J.

[b21] Gozani O, Karuman P, Jones DR, Ivanov D, Cha J, Lugovskoy AA, Baird CL, Zhu H, Field SJ, Lessnick SL, Villasenor J, Mehrotra B, Chen J, Rao VR, Brugge JS, Ferguson CG, Payrastre B, Myszka DG, Cantley LC, Wagner G, Divecha N, Prestwich GD, Yuan J (2003). The PHD finger of the chromatin-associated protein ING2 functions as a nuclear phosphoinositide receptor. Cell.

[b22] Halstead JR, Jalink K, Divecha N (2005). An emerging role for PtdIns(4,5)P_2_-mediated signalling in human disease. Trends Pharmacol Sci.

[b23] Hatten ME (1999). Central nervous system neuronal migration. Annu Rev Neurosci.

[b24] Hendry SH, Jones EG, Emson PC, Lawson DE, Heizmann CW, Streit P (1989). Two classes of cortical GABA neurons defined by differential calcium binding protein immunoreactivities. Exp Brain Res.

[b25] Hicks SW, Machamer CE (2002). The NH2-terminal domain of Golgin-160 contains both Golgi and nuclear targeting information. J Biol Chem.

[b26] Hinchliffe KA, Irvine RF, Divecha N (1998). Regulation of PtdIns4P 5-kinase C by thrombin-stimulated changes in its phosphorylation state in human platelets. Biochem J.

[b27] Hinchliffe KA, Ciruela A, Letcher AJ, Divecha N, Irvine RF (1999). Regulation of type IIalpha phosphatidylinositol phosphate kinase localisation by the protein kinase CK2. Curr Biol.

[b28] Hinchliffe KA, Giudici ML, Letcher AJ, Irvine RF (2002). Type IIalpha phosphatidylinositol phosphate kinase associates with the plasma membrane via interaction with type I isoforms. Biochem J.

[b29] Irvine RF (2003). Nuclear lipid signalling. Nat Rev Mol Cell Biol.

[b30] Ishihara H, Shibasaki Y, Kizuki N, Wada T, Yazaki Y, Asano T, Oka Y (1998). Type I phosphatidylinositol-4-phosphate 5-kinases. Cloning of the third isoform and deletion/substitution analysis of members of this novel lipid kinase family. J Biol Chem.

[b31] Itoh T, Ijuin T, Takenawa T (1998). A novel phosphatidylinositol-5-phosphate 4-kinase (phosphatidylinositol-phosphate kinase IIgamma) is phosphorylated in the endoplasmic reticulum in response to mitogenic signals. J Biol Chem.

[b32] Jentsch TJ, Hubner CA, Fuhrmann JC (2004). Ion channels: function unravelled by dysfunction. Nat Cell Biol.

[b33] Jones DR, Bultsma Y, Keune WJ, Halstead JR, Elouarrat D, Mohammed S, Heck AJ, D'Santos CS, Divecha N (2006). Nuclear PtdIns5P as a transducer of stress signaling: an in vivo role for PIP4Kbeta. Mol Cell.

[b34] Kent WJ (2002). BLAT—the BLAST-like alignment tool. Genome Res.

[b35] Koizumi S, Bootman MD, Bobanovic LK, Schell MJ, Berridge MJ, Lipp P (1999). Characterization of elementary Ca^2+^ release signals in NGF-differentiated PC12 cells and hippocampal neurons. Neuron.

[b36] Landman N, Jeong SY, Shin SY, Voronov SV, Serban G, Kang MS, Park MK, Di Paolo G, Chung S, Kim TW (2006). Presenilin mutations linked to familial Alzheimer's disease cause an imbalance in phosphatidylinositol 4,5-bisphosphate metabolism. Proc Natl Acad Sci U S A.

[b37] Lecompte O, Poch O, Laporte J (2008). PtdIns5P regulation through evolution: roles in membrane trafficking?. Trends Biochem Sci.

[b38] Lein ES, Hawrylycz MJ, Ao N, Ayres M, Bensinger A, Bernard A, Boe AF, Boguski MS, Brockway KS, Byrnes EJ, Chen L, Chen TM, Chin MC, Chong J, Crook BE, Czaplinska A, Dang CN, Datta S, Dee NR, Desaki AL, Desta T, Diep E, Dolbeare TA, Donelan MJ, Dong HW, Dougherty JG, Duncan BJ, Ebbert AJ, Eichele G, Estin LK, Faber C, Facer BA, Fields R, Fischer SR, Fliss TP, Frensley C, Gates SN, Glattfelder KJ, Halverson KR, Hart MR, Hohmann JG, Howell MP, Jeung DP, Johnson RA, Karr PT, Kawal R, Kidney JM, Knapik RH, Kuan CL, Lake JH, Laramee AR, Larsen KD, Lau C, Lemon TA, Liang AJ, Liu Y, Luong LT, Michaels J, Morgan JJ, Morgan RJ, Mortrud MT, Mosqueda NF, Ng LL, Ng R, Orta GJ, Overly CC, Pak TH, Parry SE, Pathak SD, Pearson OC, Puchalski RB, Riley ZL, Rockett HR, Rowland SA, Royall JJ, Ruiz MJ, Sarno NR, Schaffnit K, Shapovalova NV, Sivisay T, Slaughterbeck CR, Smith SC, Smith KA, Smith BI, Sodt AJ, Stewart NN, Stumpf KR, Sunkin SM, Sutram M, Tam A, Teemer CD, Thaller C, Thompson CL, Varnam LR, Visel A, Whitlock RM, Wohnoutka PE, Wolkey CK, Wong VY, Wood M, Yaylaoglu MB, Young RC, Youngstrom BL, Yuan XF, Zhang B, Zwingman TA, Jones AR (2007). Genome-wide atlas of gene expression in the adult mouse brain. Nature.

[b39] Lorenz H, Hailey DW, Wunder C, Lippincott-Schwartz J (2006). The fluorescence protease protection (FPP) assay to determine protein localization and membrane topology. Nat Protoc.

[b40] Madalosso SH, Perez-Villegas EM, Armengol JA (2005). Naturally occurring neuronal death during the postnatal development of Purkinje cells and their precerebellar afferent projections. Brain Res Brain Res Rev.

[b41] Merlot S, Meili R, Pagliarini DJ, Maehama T, Dixon JE, Firtel RA (2003). A PTEN-related 5-phosphatidylinositol phosphatase localized in the Golgi. J Biol Chem.

[b42] Morris JB, Hinchliffe KA, Ciruela A, Letcher AJ, Irvine RF (2000). Thrombin stimulation of platelets causes an increase in phosphatidylinositol 5-phosphate revealed by mass assay. FEBS Lett.

[b43] Nakano-Kobayashi A, Yamazaki M, Unoki T, Hongu T, Murata C, Taguchi R, Katada T, Frohman MA, Yokozeki T, Kanaho Y (2007). Role of activation of PIP5Kgamma661 by AP-2 complex in synaptic vesicle endocytosis. EMBO J.

[b44] Parkinson H, Kapushesky M, Shojatalab M, Abeygunawardena N, Coulson R, Farne A, Holloway E, Kolesnykov N, Lilja P, Lukk M, Mani R, Rayner T, Sharma A, William E, Sarkans U, Brazma A (2007). ArrayExpress—a public database of microarray experiments and gene expression profiles. Nucleic Acids Res.

[b45] Pelkmans L, Fava E, Grabner H, Hannus M, Habermann B, Krausz E, Zerial M (2005). Genome-wide analysis of human kinases in clathrin- and caveolae/raft-mediated endocytosis. Nature.

[b46] Pendaries C, Tronchere H, Racaud-Sultan C, Gaits-Iacovoni F, Coronas S, Manenti S, Gratacap MP, Plantavid M, Payrastre B (2005). Emerging roles of phosphatidylinositol monophosphates in cellular signaling and trafficking. Adv Enzyme Regul.

[b47] Polleux F, Whitford KL, Dijkhuizen PA, Vitalis T, Ghosh A (2002). Control of cortical interneuron migration by neurotrophins and PI3-kinase signaling. Development.

[b48] Quinn B, Toga AW, Motamed S, Merlic CA (1995). Fluoro Nissl green: a novel fluorescent counterstain for neuroanatomy. Neurosci Lett.

[b49] Rameh LE, Tolias KF, Duckworth BC, Cantley LC (1997). A new pathway for synthesis of phosphatidylinositol-4,5-bisphosphate. Nature.

[b50] Richardson JP, Wang M, Clarke JH, Patel KJ, Irvine RF (2007). Genomic tagging of endogenous type IIbeta phosphatidylinositol 5-phosphate 4-kinase in DT40 cells reveals a nuclear localisation. Cell Signal.

[b51] Sarafian V, Jadot M, Foidart JM, Letesson JJ, Van den Brule F, Castronovo V, Wattiaux R, Coninck SW (1998). Expression of Lamp-1 and Lamp-2 and their interactions with galectin-3 in human tumor cells. Int J Cancer.

[b52] Sbrissa D, Ikonomov OC, Strakova J, Shisheva A (2004). Role for a novel signaling intermediate, phosphatidylinositol 5-phosphate, in insulin-regulated F-actin stress fiber breakdown and GLUT4 translocation. Endocrinology.

[b53] Schleiermacher G, Bourdeaut F, Combaret V, Picrron G, Raynal V, Aurias A, Ribeiro A, Janoueix-Lerosey I, Delattre O (2005). Stepwise occurrence of a complex unbalanced translocation in neuroblastoma leading to insertion of a telomere sequence and late chromosome 17q gain. Oncogene.

[b54] Suh BC, Hille B (2005). Regulation of ion channels by phosphatidylinositol 4,5-bisphosphate. Curr Opin Neurobiol.

[b55] van Horck FP, Lavazais E, Eickholt BJ, Moolenaar WH, Divecha N (2002). Essential role of type I(alpha) phosphatidylinositol 4-phosphate 5-kinase in neurite remodeling. Curr Biol.

[b56] Voronov SV, Frere SG, Giovedi S, Pollina EA, Borel C, Zhang H, Schmidt C, Akeson EC, Wenk MR, Cimasoni L, Arancio O, Davisson MT, Antonarakis SE, Gardiner K, De Camilli P, Di Paolo G (2008). Synaptojanin 1-linked phosphoinositide dyshomeostasis and cognitive deficits in mouse models of Down's syndrome. Proc Natl Acad Sci U S A.

[b57] Wang Y, Lian L, Golden JA, Morrisey EE, Abrams CS (2007). PIP5KI gamma is required for cardiovascular and neuronal development. Proc Natl Acad Sci U S A.

[b58] Wenk MR, Pellegrini L, Klenchin VA, Di Paolo G, Chang S, Daniell L, Arioka M, Martin TF, De Camilli P (2001). PIP kinase Igamma is the major PI(4,5)P(2) synthesizing enzyme at the synapse. Neuron.

[b59] Wilcox A, Hinchliffe KA (2008). Regulation of extranuclear PtdIns5P production by phosphatidylinositol phosphate 4-kinase 2alpha. FEBS Lett.

[b60] Xie Y, Ding YQ, Hong Y, Feng Z, Navarre S, Xi CX, Zhu XJ, Wang CL, Ackerman SL, Kozlowski D, Mei L, Xiong WC (2005). Phosphatidylinositol transfer protein-alpha in netrin-1-induced PLC signalling and neurite outgrowth. Nat Cell Biol.

[b61] Yin HL, Janmey PA (2003). Phosphoinositide regulation of the actin cytoskeleton. Annu Rev Physiol.

[b62] Zou J, Marjanovic J, Kisseleva MV, Wilson M, Majerus PW (2007). Type I phosphatidylinositol-4,5-bisphosphate 4-phosphatase regulates stress-induced apoptosis. Proc Natl Acad Sci U S A.

