# Supplementary figures and images for: Distribution and neuronal expression of phosphatidylinositol phosphate kinase IIγ in the mouse brain
Source: J Comp Neurol. 2009 Jul 16;517(3):296–312. doi: 10.1002/cne.22161 (PMC2814081; doi:10.1002/cne.22161)

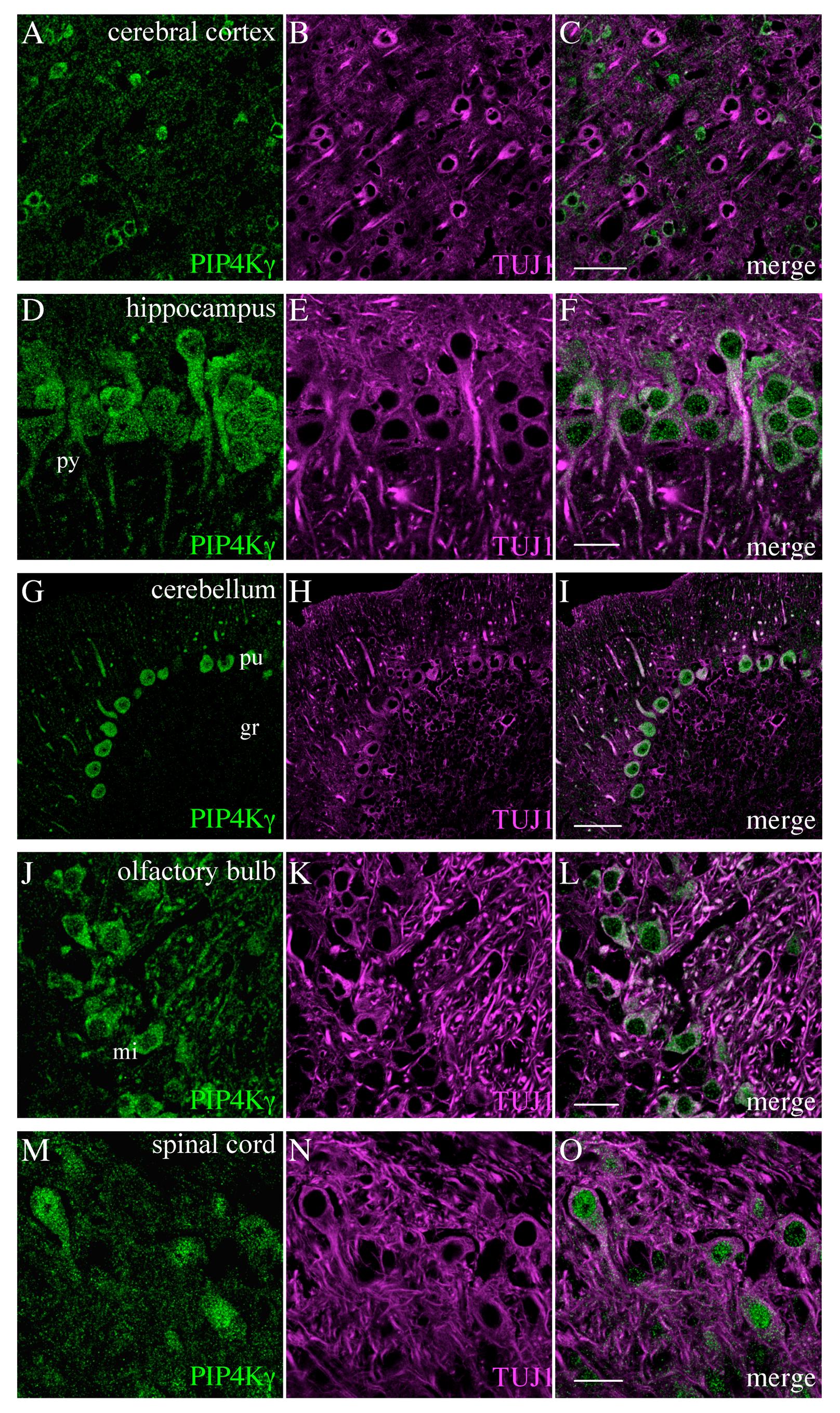

Supplement: Supplementary file 1 [file cne0517-0296-SD1.tif]

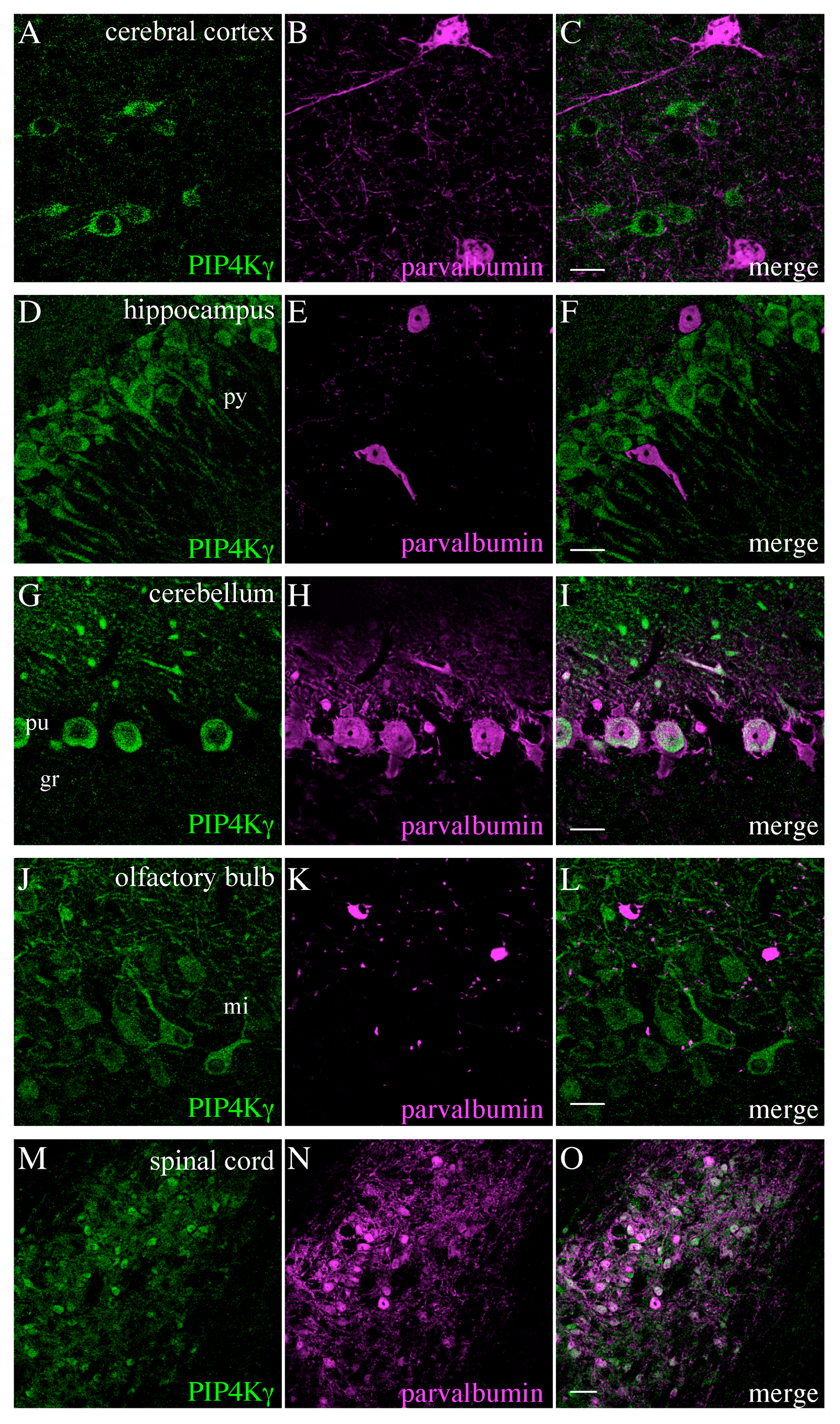

Supplement: Supplementary file 2 [file cne0517-0296-SD2.tif]

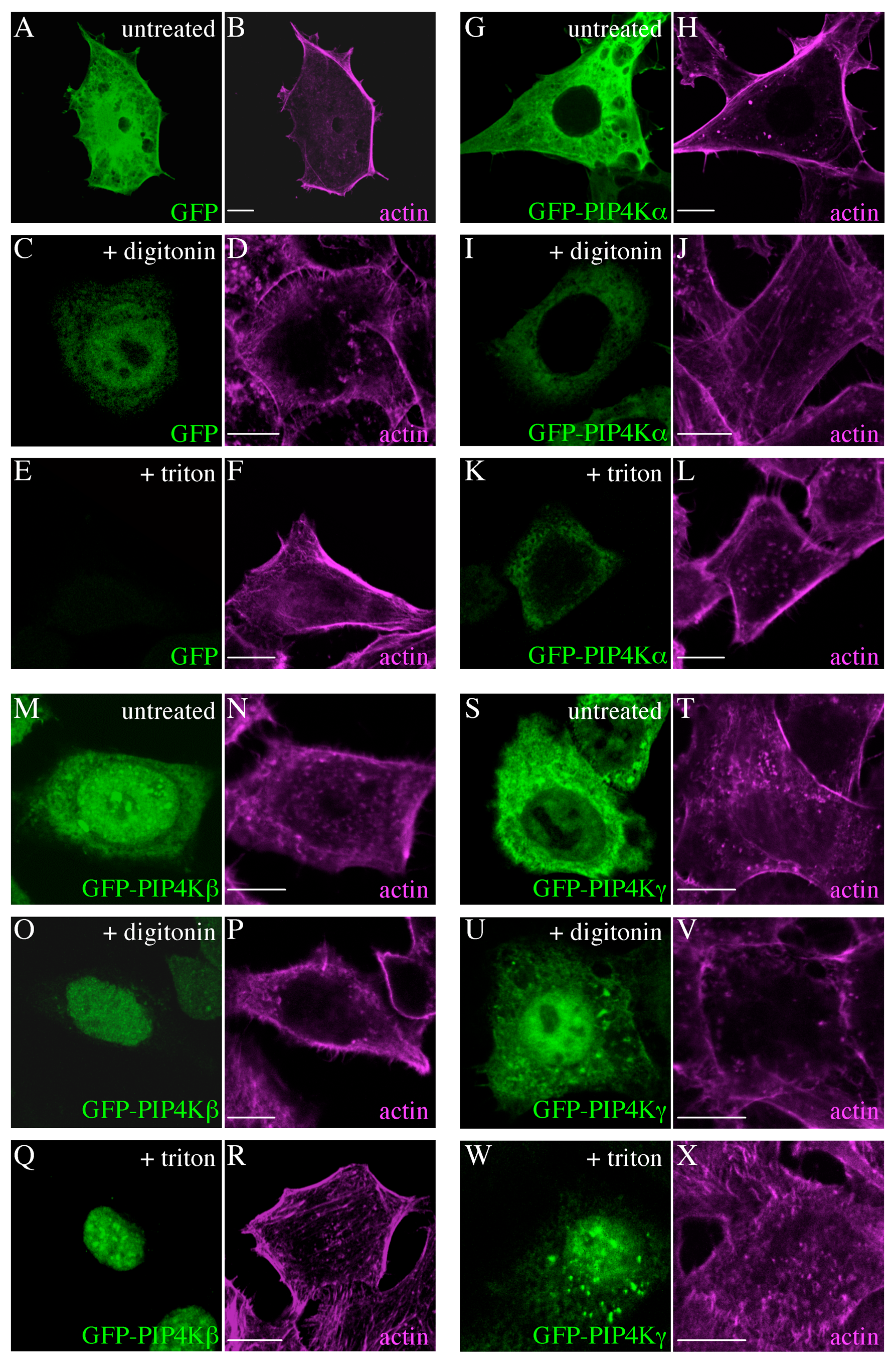

Supplement: Supplementary file 3 [file cne0517-0296-SD3.tif]

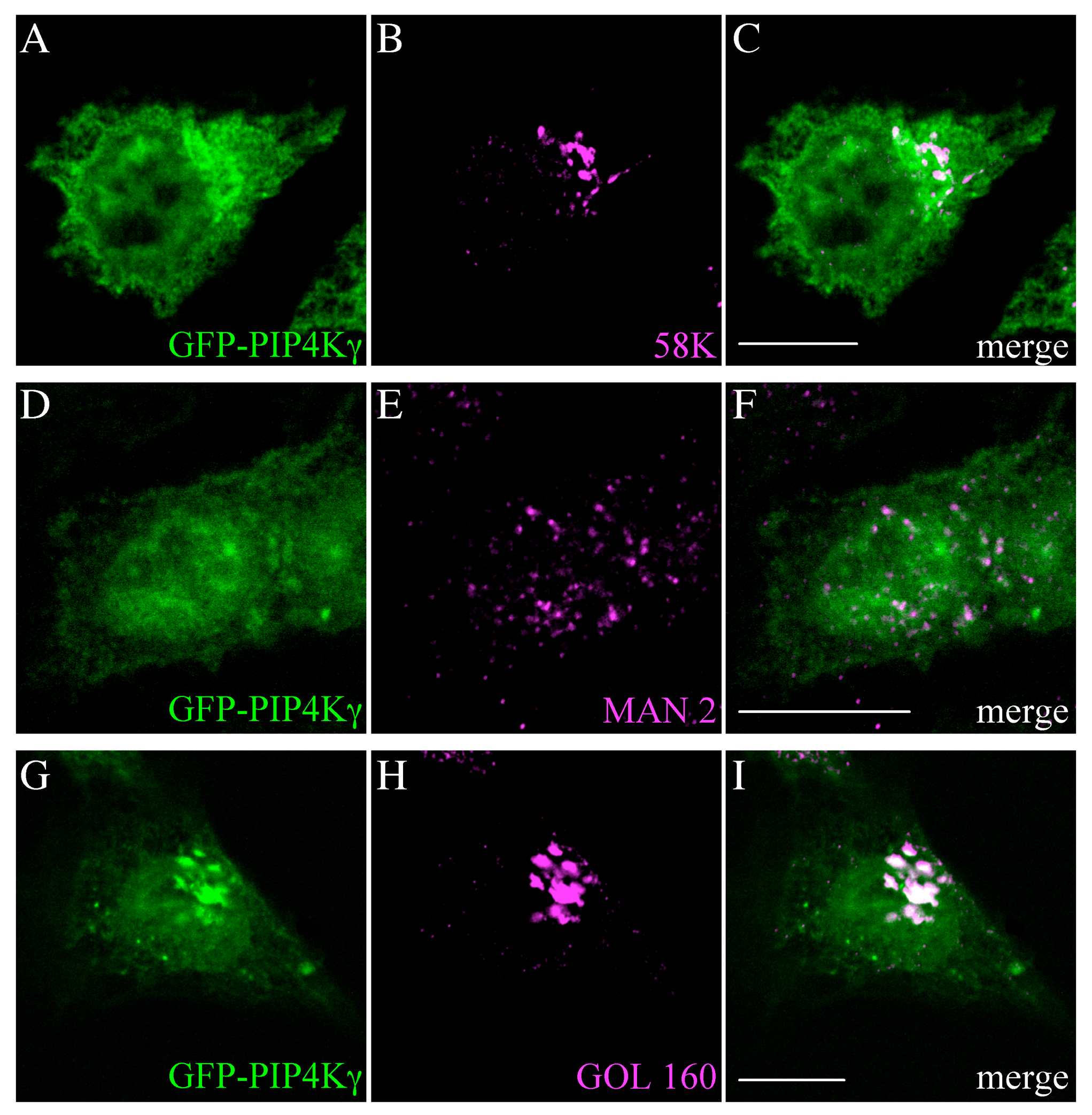

Supplement: Supplementary file 4 [file cne0517-0296-SD4.tif]

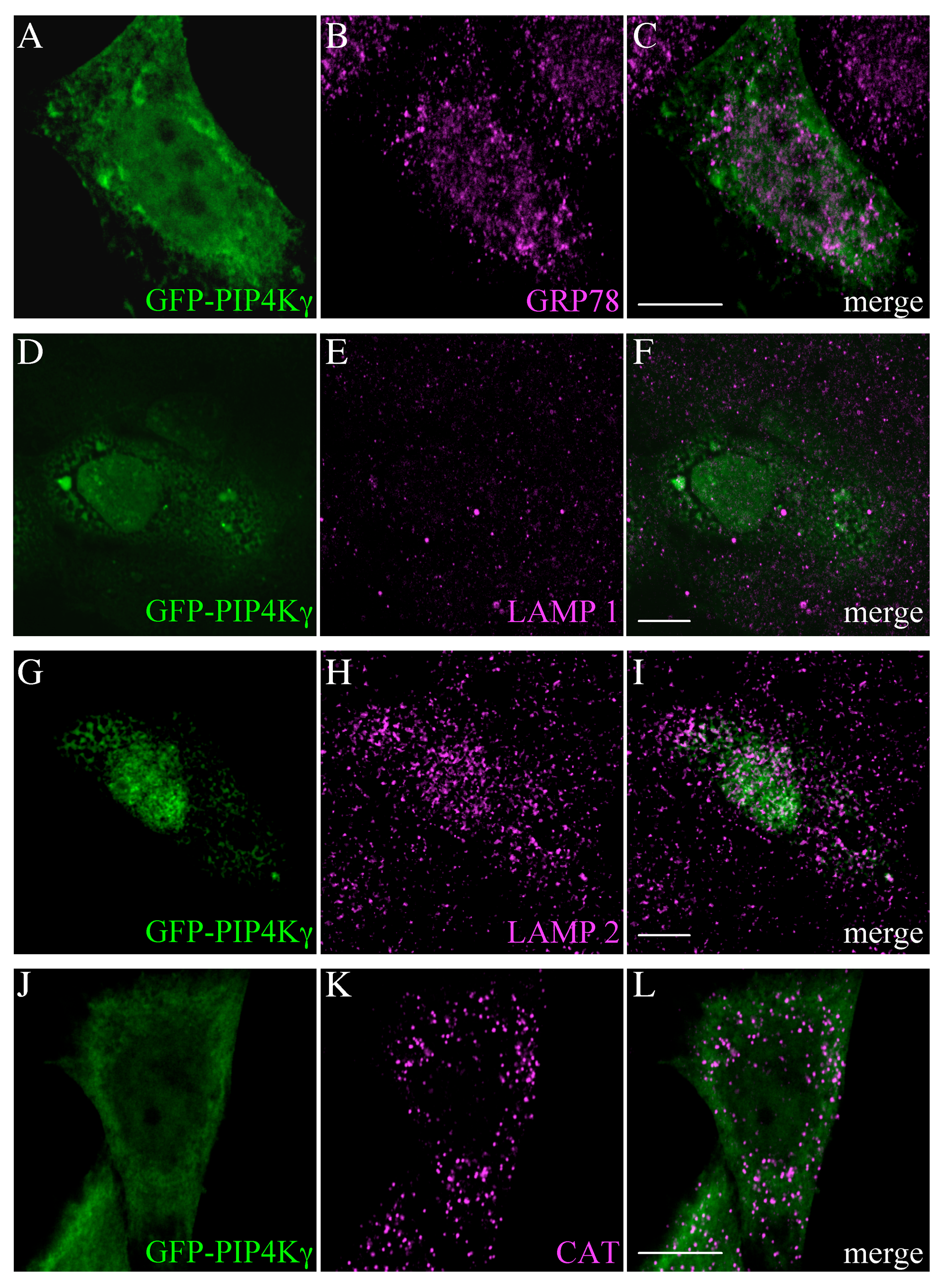

Supplement: Supplementary file 5 [file cne0517-0296-SD5.tif]

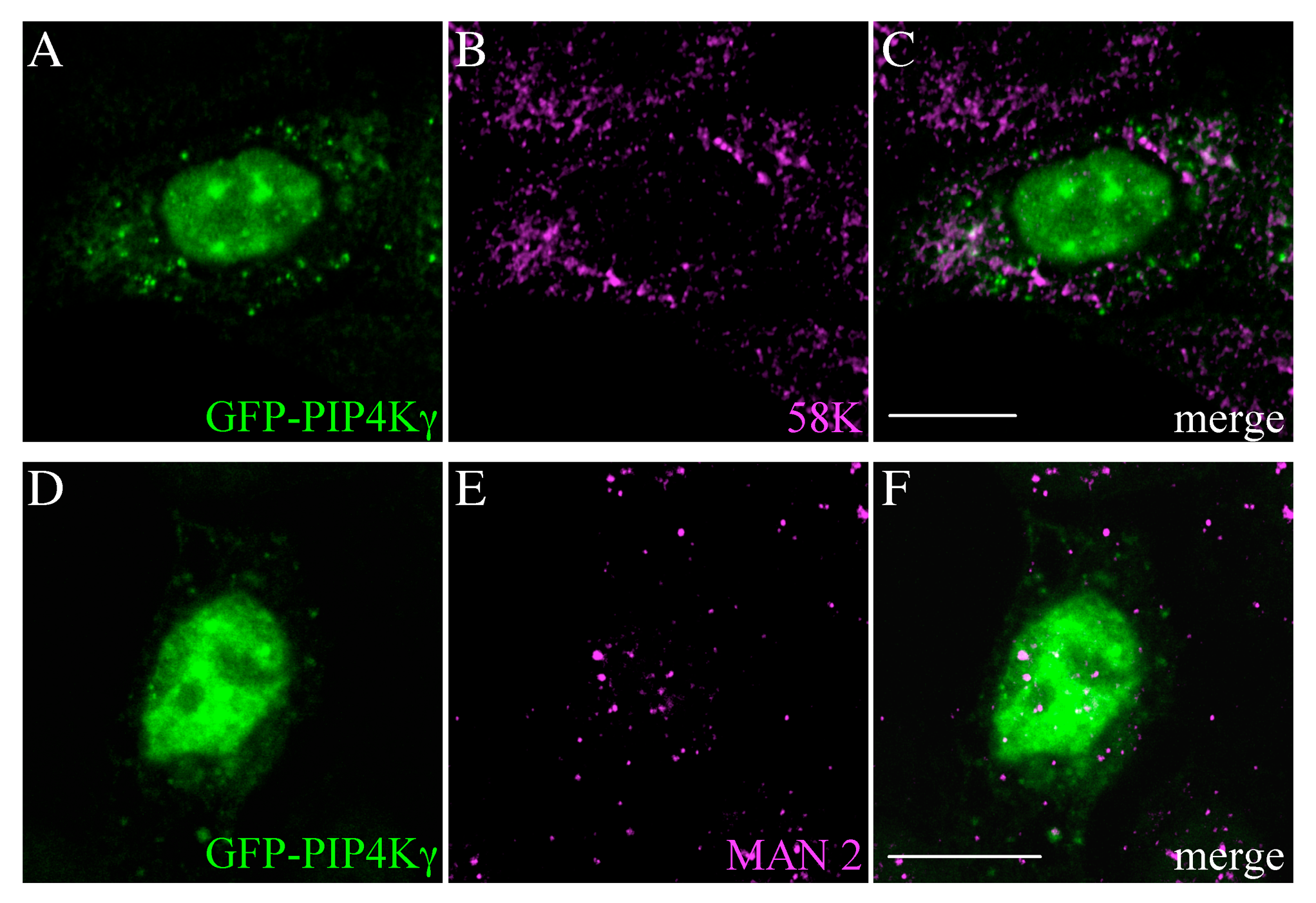

Supplement: Supplementary file 6 [file cne0517-0296-SD6.tif]
